# Multifunctional Biomimetic Nanocarriers for Dual‐Targeted Immuno‐Gene Therapy Against Hepatocellular Carcinoma

**DOI:** 10.1002/advs.202400951

**Published:** 2024-07-08

**Authors:** Yupeng Sun, Yan Liu, Rui Li, Cuilin Zhang, Ming Wu, Xiaolong Zhang, Aixian Zheng, Naishun Liao, Youshi Zheng, Haipo Xu, Rui Zeng, Yongyi Zeng, Xiaolong Liu

**Affiliations:** ^1^ The United Innovation of Mengchao Hepatobiliary Technology Key Laboratory of Fujian Province Mengchao Hepatobiliary Hospital of Fujian Medical University Fuzhou 350025 P. R. China; ^2^ Mengchao Med‐X Center Fuzhou University Fuzhou 350116 P. R. China; ^3^ College of Biological Science and Engineering Fuzhou University Fuzhou 350116 P. R. China; ^4^ Liver Disease Center The First Affiliated Hospital of Fujian Medical University Fuzhou 350005 P. R. China; ^5^ CAS Key Laboratory of Design and Assembly of Functional Nanostructures Fujian Institute of Research on the Structure of Matter Chinese Academy of Sciences Fuzhou 350002 P. R. China

**Keywords:** immune checkpoint blockade, immuno‐gene therapy, immuno‐sensitization, multifunctional biomimetic nanocarrier, PDL1/Pbrm1 gene

## Abstract

Growing evidences have proved that tumors evade recognition and attack by the immune system through immune escape mechanisms, and PDL1/Pbrm1 genes have a strong correlation with poor response or resistance to immune checkpoint blockade (ICB) therapy. Herein, a multifunctional biomimetic nanocarrier (siRNA‐CaP@PD1‐NVs) is developed, which can not only enhance the cytotoxic activity of immune cells by blocking PD1/PDL1 axis, but also reduce tumor immune escape via Pbrm1/PDL1 gene silencing, leading to a significant improvement in tumor immunosuppressive microenvironment. Consequently, the nanocarrier promotes DC cell maturation, enhances the infiltration and activity of CD8+ T cells, and forms long‐term immune memory, which can effectively inhibit tumor growth or even eliminate tumors, and prevent tumor recurrence and metastasis. Overall, this study presents a powerful strategy for co‐delivery of siRNA drugs, immune adjuvant, and immune checkpoint inhibitors, and holds great promise for improving the effectiveness and safety of current immunotherapy regimens.

## Graphical abstract

A multi‐functional biomimetic nanocarrier (siRNA‐CaP@PD1‐NVs) is developed, capable of simultaneous and efficient delivery of nucleic acid drugs (siRNA), immune adjuvant (CaP), and quasi immune checkpoint inhibitors (PD1‐NVs). It can not only enhance the cytotoxic activity of immune cells by blocking PD1/PDL1 axis, but also reduce immune escape via Pbrm1/PDL1 gene silencing, leading to a significant improvement in tumor immuno‐gene therapy.

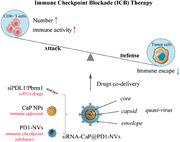

## 1 Introduction

Immunotherapy is gaining momentum as a promising therapeutic approach for cancer, in which, immune cells are able to identify and eliminate cancer cells by initiating the cancer‐immune cycle.^[^ ^1^ ^]^ Especially, immune checkpoint blockade (ICB) therapy targeting PD1/PDL1 axis has demonstrated excellent clinical benefits as the standard of care for numerous cancers. However, the overall response rates for solid tumors are relatively low (less than 30%),^[^ ^2^ ^]^ most likely due to the significant upregulation of endogenous PDL1 mRNA expression, which induces the intracellular PDL1 to return back to the surface of tumor cells, to maintain a homeostasis state and mediate immune resistance.^[^ ^3^ ^]^ Thus, it may not be sufficient for targeting a single signaling pathway to attain robust anti‐tumor immunity and substantial therapeutic benefits.

Several strategies have been proposed to improve the efficacy of immune checkpoint blocking therapy, such as CuPP nanoenzymes‐driven macrophage reprogramming combined with PDL1 antibody,^[^ ^4^ ^]^ and the combination of reactivation of the tumor suppressor PTEN with ICB therapy.^[^ ^5^ ^]^ Although these strategies have achieved excellent tumor therapeutic effects, due to the complexity of tumor microenvironment, the diversity of cancer types, and individual differences, tumor cells still have the ability to evade the immune system via various immune escape mechanisms,^[^ ^6^ ^]^ such as recruiting immunosuppressive cells, upregulating immunosuppressive molecules and disturbing immunostimulatory cytokines or metabolite, which result in immune insensitivity of tumor cells and limiting therapeutic outcomes.^[^ ^7^ ^]^ Recently, Eliezer et al. revealed that the patients with Pbrm1 gene loss experienced significant improvement in the effectiveness of ICB therapy.^[^ ^8^ ^]^ Pbrm1 plays a crucial role in resisting T cell attacks, and Pbrm1 deficiency can alter the expression of genes within tumor cells, rendering them more sensitive to immune attack and preventing immune evasion.^[^ ^9^ ^]^ Therefore, Pbrm1 can serve as a potential target to develop innovative strategies to enhance the clinical benefit of ICB therapy.

Here, we propose a dual‐targeted immuno‐gene therapy, by silencing the immunosuppressive gene (PDL1) and immuno‐sensitizing gene (Pbrm1) via small interfering RNA (siRNA), to prevent immune evasion and improve immune responses. It was reported that nano‐carriers such as lipid nanoparticles (LNPs), hydrogels, or meso‐porous silicon can be utilized to deliver nucleic acid drugs.^[^ ^10^ ^]^ However, therapeutic siRNA/mRNA endures short half‐life, poor tumor‐specificity, or low intracellular delivery efficiency, limiting its clinical translation and application.^[^ ^11^ ^]^

Calcium phosphate (CaP) has emerged as a promising non‐viral vector in gene therapy, due to its excellent biocompatibility and biodegradability.^[^ ^12^ ^]^ The phosphate group of nucleic acids interacts with calcium ions, allowing for efficient and complete encapsulation of nucleic acids. Moreover, CaP displays a pH‐sensitive property, enabling it to rapidly dissolve in lysosomes and release its cargo into the cytoplasm.^[^ ^13^ ^]^ Besides, calcium ions (Ca^2+^) act as second messengers in many cell types, including lymphocytes, and the intracellular Ca^2+^ signaling can affect immune cell functions such as metabolism, proliferation, differentiation, secretion of antibodies or cytokines, and cytotoxicity.^[^ ^14^ ^]^ However, it still faces challenges in maintaining stability in vivo circulation and facilitating effective tumor enrichment. In recent studies, biomimetic nanoparticles based on cell membranes have exhibited prolonged circulation and exceptional tumor‐targeting capability in vivo.^[^ ^15^ ^]^ For instance, cancer cell and immune cell membranes have been proposed with unique tumor‐homing ability and prolonged blood circulation time.^[^ ^16^ ^]^ Tumor‐antigen activated dendritic cell membrane (aDCM) can enhance homotypic‐targeting and blood‐brain barrier (BBB)‐crossing efficiently and improve the immune response.^[^ ^17^ ^]^ Genetically engineered cell membrane can hold the ability to extend the in vivo residence times, target specific cells, tissue, or organs, and regulate the immunosuppressive microenvironment by a clever design, potentially enhancing the efficacy of immune checkpoint blockade (ICB) therapy.^[^ ^18^ ^]^ Besides, our group has designed a novel nanovesicles decorated with PD1 protein (PD1‐NVs), which not only disrupt the PD1/PDL1 signal path, but also target tumor via the specific binding between the PD1 protein on PD1‐NVs and PDL1 ligand on tumor cells.^[^ ^19^ ^]^

**Scheme 1:**
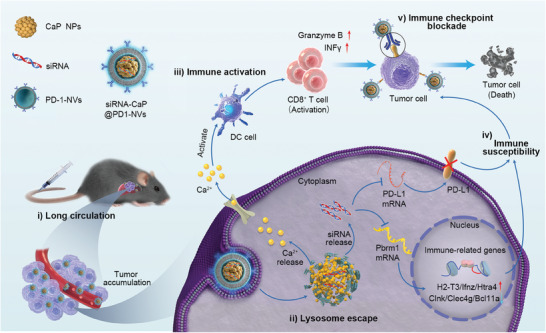
Schematic illustration of multifunctional biomimetic nanocarriers for dual‐targeted immuno‐gene therapy. A virus‐like multifunctional nanocarrier (siRNA‐CaP@PD1‐NVs) is fabricated by siRNA drugs, immune adjuvant (CaP), and immune checkpoint inhibitor (PD1‐NVs), which enables efficient co‐delivery via the excellent capabilities of long circulation and lysosomes escape. It can not only initiate T cell‐mediated immune responses, but also enhance the sensitivity of cancer cells, ultimately improving immune checkpoint blockade therapy.

In this study, we designed a virus‐like multifunctional nanocarrier (siRNA‐CaP@PD1‐NVs) to harbor nucleic acids for immuno‐gene therapy, in which, siRNA of both PDL1/Pbrm1 acts as the “viral core”, calcium phosphate (CaP) acts as the “viral capsid” and PD1 protein‐decorated cell membrane serves as the “viral envelope” (**Scheme** **1**). This virus‐like nanocarrier exhibits multifunctional characteristics, including (i) PD1‐NVs decoration increases the stability of the nanocarrier that enhances the circulation time in vivo and facilitates their tumor accumulation; (ii) a pH stimuli‐responsive CaP core, not only effectively loads nucleic acid drugs to avoid degradation by nucleases, but also allows for the rupture of lysosomes, increasing the cytoplasmic delivery of siRNA; (iii) calcium ions (Ca^2+^) can serve as immune adjuvants to activate dendritic cells (DCs) and initiate T cell‐mediated immune responses; (iv) both PDL1/Pbrm1 gene silencing enhances the sensitivity of tumor cells to immune therapy, preventing tumor immune evasion; and (v) PD1‐NVs can also serve as an immune checkpoint inhibitor to block the PD1/PDL1 axis, enhancing the cytotoxic activity of effector T cells. Therefore, the biomimetic nanocarrier (siRNA‐CaP@PD1‐NVs), which enables efficient co‐delivery of siRNA drugs, immune adjuvant (CaP), and the immune checkpoint inhibitor (PD1‐NVs), holds great potential in developing effective and safe immunotherapy regimens.

## 2 Results and Discussion

### 2.1 Synthesis and Characterization of siRNA‐CaP@PD1‐NVs

**Figure 1:**
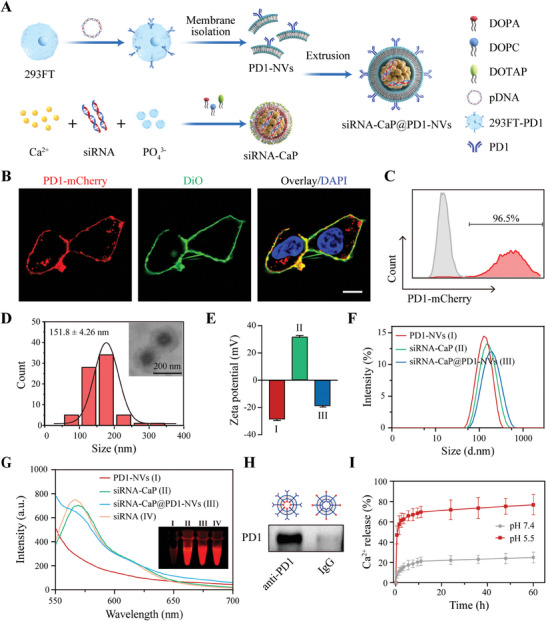
Synthesis and characterization of siRNA‐CaP@PD1‐NVs. A) Schematic illustration of the synthesis process of siRNA‐CaP@PD1‐NVs. B) Confocal images of HEK293FT cells stably expressing fusion protein PD1‐mCherry (red) on cell membrane, cell membrane was stained by DiO (green). Scale bar:10 µm. C) The representative FCM profiles of PD1‐expressing HEK293FT stable cells (gated by mCherry^+^). D) The size distribution of siRNA‐CaP@PD1‐NVs and the representative TEM image (insert picture). E) Zeta potential and F) Hydrodynamic size distribution of PD1‐NVs, siRNA‐CaP, siRNA‐CaP@PD1‐NVs. Data are presented as mean ± SD (*n* = 3). G) UV/vis absorption spectra of free siRNA, PD1‐NVs, siRNA‐CaP, siRNA‐CaP@PD1‐NVs, inset: the fluorescence spectra under 555 nm excitation. H) Immunoprecipitation and western blot analysis to verify the right orientation of PD‐1 on the siRNA‐CaP@PD1‐NVs. anti‐PD1 antibody was used to bind to PD1 protein and pull it down from siRNA‐CaP@PD1‐NVs. I) The cumulative release profiles of Ca^2+^ from siRNA‐CaP@PD1‐NVs incubated at different pH values (5.5 or 7.4, Data are presented as mean ± SD, *n* = 3).

Considering the circulating stability in vivo and tumor enrichment capacity of biomimetic nanovesicles, as illustrated in **Figure** **1**A, HEK 293FT cells were selected to prepare the biomimetic nanovesicles decorated with PD1 proteins (PD1‐NVs) by gene engineering. The PD1 proteins (fusing with mCherry) were located on the cell membrane of HEK 293FT cells (stained by DiO), preliminarily indicated the successful expression of PD1 proteins on HEK 293FT cells (Figure 1B). Subsequently, flow cytometry (FCM) analysis showed that ≈96.5% of HEK 293FT cells expressed the PD1‐mCherry fusion proteins (Figure 1C). Furthermore, the over‐expressing PD1 proteins could also be verified by western blot (WB) assay (Figure S1, Supporting Information), and the proportion of PD1 protein to total membrane proteins was 3.52%, detected by enzyme‐linked immunosorbent assay (ELISA) (Figure S2, Supporting Information). Taking together, the above results confirmed the successful expressing of PD1 proteins on the membrane of genetically engineered HEK 293FT cells.

The siRNA was encapsulated in the core of calcium phosphate nanoparticles (CaP NPs), which were synthesized via a modified microemulsion method, using calcium chloride (CaCl_2_) as the calcium source, disodium hydrogen phosphate (Na_2_HPO_4_) as the phosphorus source and dioleyl phosphate (DOPA) as the surfactant (Figure 1A). Then, the cell membranes with the over‐expressing PD1 proteins were isolated from genetically engineered HEK 293FT cells, and coated on the surface of siRNA‐CaP by extrusion method, to generate siRNA‐CaP@PD1‐NVs.

Transmission electron microscopy (TEM) images showed that the average diameter of siRNA‐CaP and siRNA‐CaP@PD1‐NVs was 98.3 ± 10.55 nm and 151.8 ± 4.26 nm, respectively. Meanwhile, the siRNA‐CaP@PD1‐NVs displayed a virus‐like morphology with a CaP core and an outer membrane structure. (Figure 1D; Figure S3, Supporting Information). As shown in Figure 1E, the zeta potential of siRNA‐CaP@PD1‐NVs was significantly changed from +31.3 mV (siRNA‐CaP) to −18.5 mV, possibly due to the surface coating of purified PD1‐NVs (−28.3 mV). Dynamic light scattering (DLS) demonstrated that the hydrodynamic size of siRNA‐CaP@PD1‐NVs was ≈168.6 ± 2.48 nm with polydispersityindex (PDI) of 0.2 (Figure 1F), and which was unchanged significantly under physiological conditions (pH 7.4) for 72 h (Figure S4, Supporting Information), indicating a good water‐dispersity and remarkable stability, which is beneficial to its biological application in vivo.

To confirm the successful loading of siRNA in the CaP NPs, Alexa Fluor 555 labeled siRNA was employed to fabricate siRNA‐CaP@PD1‐NVs. UV/vis absorption spectra showed that the characteristic peak (at 565 nm) of siRNA‐CaP was similar to that of free siRNA (at 562 nm), after coating the cell membrane, there were no obvious influences on its characteristic absorption. Additionally, the fluorescence images demonstrated representative signals of Alexa Fluor 555 dye for siRNA‐CaP, siRNA‐CaP@PD1‐NVs, and free siRNA (Figure 1G). The aforementioned results indicated the successful encapsulation of siRNA into CaP NPs and the loading efficiency of siRNA was 75.42 ± 1.28% (Figure S5, Supporting Information). Next, the protein pattern of siRNA‐CaP@PD1‐NVs were similar to the purified PD1‐NVs, measured by coomassie blue staining (Figure S6, Supporting Information), indicating the successful coating of PD1‐NVs on siRNA‐CaP. Furthermore, the PD1 proteins were pulled down from the siRNA‐CaP@PD1‐NVs by anti‐PD1 antibody which bond to the magnetic beads modified with the protein A/G, and detected by the immunoprecipitation assay. As illustrated in Figure 1H, the characteristic band of PD1 protein with molecular weight of ≈92 kDa was observed in anti‐PD1 group, indicating a right outside‐out orientation, which assisted in the immune checkpoint blockade function of siRNA‐CaP@PD1‐NVs.

To evaluate the acid‐responsive dissociation ability of siRNA‐CaP@PD1‐NVs, the in vitro release patterns of Ca^2+^ were monitored under different pH conditions (5.5 or 7.4). The released percentage of Ca^2+^ from siRNA‐CaP@PD1‐NVs was only 22.06 ± 2.371% under physiological conditions (pH 7.4) after 72 h incubation (Figure 1I). Furthermore, a higher Ca^2+^ release (72.97 ± 3.260%) was detected in a simulated lysosomal environment (pH 5.5). In addition, TEM further confirmed that the siRNA‐CaP@PD1‐NVs was degraded and generated numerous smaller‐sized nanoparticles at pH 5.5 (Figure S7, Supporting Information). These results indicated that the release of Ca^2+^ from siRNA‐CaP@PD1‐NVs was pH dependent, in which hydrion (H^+^) can penetrate the cell membrane barrier to trigger the hydrolysis of the siRNA‐CaP cores in an acidic microenvironment, thereby contributing the lysosomal escape of siRNA and reducing the off‐target release in physiological circumstances.^[^ ^20^ ^]^

### 2.2 Multifunctional Validation of siRNA‐CaP@PD1‐NVs In Vitro

The cytotoxicity of siRNA‐CaP@PD1‐NVs was evaluated by cell counting kit‐8 (CCK‐8) assay. It was revealed that approximately 90% of hepa1‐6 cells kept good cell viability after incubating with siRNA‐CaP@PD1‐NVs for 24 h, in sharp contrast to those cells treated by siRNA‐CaP (Figure S8, Supporting Information), preliminarily indicating a good bio‐safety of siRNA‐CaP@PD1‐NVs.

#### 2.2.1 siRNA‐CaP@PD1‐NVs‐Mediated Efficient Intracellular Delivery of siRNA

**Figure 2:**
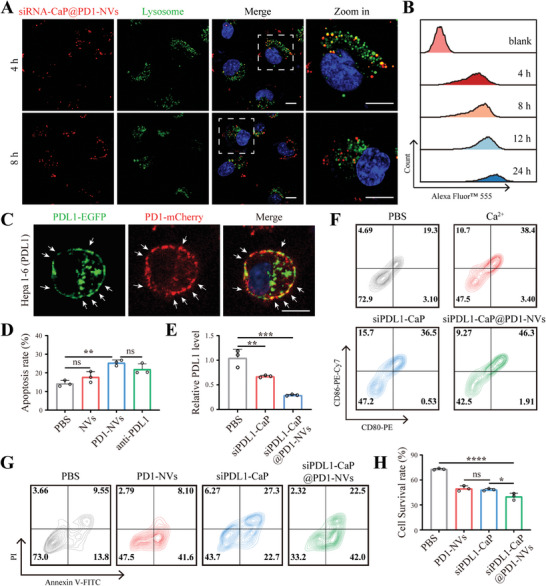
A) Lysosome escape of siRNA (labeled by Alexa Fluor 555, red) loaded by siRNA‐CaP@PD1‐NVs after incubation for 4 and 8 h in Hepa 1–6 cells, scale bar: 10 µm. Nucleus were stained with Hoechst 33 342 (blue) and lysosomes were indicated by LysoTracker Green. B) The representative FCM profiles of Hepa1‐6 cells treated with siRNA‐CaP@PD1‐NVs for different incubation times. C) Fluorescence images of Hepa 1–6 cells (PDL1‐EGFP) incubated with PD1‐NVs (PD1‐mCherry) for 2 h at 4 °C, scale bar: 10 µm. D) Apoptosis rate of Hepa 1–6 cells treated with PBS, pure nanovesicles (NVs), PD1‐NVs, or PDL1 antibody (anti‐PDL1) after co‐incubation with activated CD8+T cells for 24 h detected by LDH assay. ns: not significant, *p* > 0.05, **p < 0.01, *n* = 3, one‐way ANOVA followed by Tukey's multiple comparison test. E) PDL1 gene expression in Hepa 1–6 cells incubated with PBS, siPDL1‐CaP, and siPDL1‐CaP@PD1‐NVs for 24 h, respectively. **p < 0.01, ***p < 0.001, *n* = 3, one‐way ANOVA followed by Tukey's multiple comparison test. F) Flow cytometric profiles of CD80 and CD86 on DCs after co‐incubation with PBS, Ca^2+^, siRNA‐CaP, and siRNA‐CaP@PD1‐NVs for 48 h, respectively. G) Flow cytometry profiles of Hepa 1–6 cells with indicated treatments after co‐incubation with activated CD8+T cells for 24 h, staining with Annexin V‐FITC/PI, and H) the statistical analysis of cell survival rate. (ns: not significant, *p* > 0.05, *p < 0.05, ****p < 0.0001, *n* = 3, one‐way ANOVA followed by Tukey's multiple comparison test).

It's important for gene therapy to efficiently deliver siRNA into cytoplasm, as siRNA needs to recognize and bind to mRNA for gene silencing.^[^ ^21^ ^]^ Non‐viral siRNA delivery faces a significant hurdle in achieving endosomal escape, due to acidic microenvironment and multiple nucleases in lysosomes.^[^ ^22^ ^]^ To evaluate the lysosome escape ability of siRNA‐CaP@PD1‐NVs, we observed the co‐localization between siRNA‐CaP@PD1‐NVs (siRNA, labeled by Alexa Fluor 555, red) and lysosome (indicated by LysoTracker Green) at different time points by confocal laser microscopy (CLSM). As illustrated in **Figure**. **2**A, there were a large amount of fluorescent co‐localization signals in Hepa1‐6 cells incubated with siRNA‐CaP@PD1‐NVs for 4 h, as the incubation time increased, it was observed that the siRNA successfully exited the lysosomes and dispersed into the cytoplasm of Hepa1‐6 cells after incubating for 8 h. Moreover, the colocalization coefficients of siRNA and lysosomes at 4 h or 8 h were 0.72 ± 0.18 and 0.44 ± 0.12, respectively (Figure S9, Supporting Information), suggesting the efficient lysosomal escape of siRNA. Therefore, we speculate that the CaP core of siRNA‐CaP@PD1‐NVs would rapidly decompose into calcium and phosphate ions in the acidic environment of lysosomes, which increases the osmotic pressure of lysosomes, leading to the influx of water molecules and osmotic swelling of the lysosomes. Eventually, the lysosomes burst and release the siRNA, calcium and phosphate ions into the cytoplasm.^[^ ^10^, ^13^, ^23^ ^]^ At the same time, the siRNA‐CaP@PD1‐NVs could also induce the formation of autophagosomes to redeliver them into lysosomes for degradation, further promoting lysosome escape of siRNA (Figure S10, Supporting Information).

In addition, the cell uptake rate and average fluorescence intensity gradually increased, and the cell uptake rate approached ≈100% after co‐incubating for 12 h (**Figure** **2**B; Figure S11, Supporting Information). Collectively, these findings indicated a high efficiency of siRNA‐CaP@PD1‐NVs in facilitating cytosol siRNA release.

#### 2.2.2 Functional Validation of Immune Checkpoint Blockade

It is well known that the highly expressed PDL1 on cancer cells can bind with PD1 on TILs, to inhibit the activity of TILs, resulting in immune escape of tumor cells. In clinical practice, anti‐PDL1, as an immune checkpoint inhibitor, was used to block PD1/PDL1 axis to reverse T cell activity, and improve therapeutic efficacy of tumor immunotherapy.^[^ ^24^ ^]^

To investigate the immune checkpoint blockade function of siRNA‐CaP@PD1‐NVs, first, PD1‐NVs (with mCherry tag) were added into the Hepa1‐6 cells (over‐expressing PDL1‐EGFP) and incubating for 2 h at 4 °C, the significant fluorescence co‐localization signals between PDL1‐EGFP (green) and PD1‐mCherry (red) was observed by CLSM (Figure 2C), implying the binding of PD1‐NVs with PDL1 in Hepa1‐6 cells. To further verify that the specific binding was mediated by the interaction between PD1 and PDL1, hepa1‐6 cells were pre‐treated with anti‐PDL1 antibody and then incubated with DiI‐modified PD1‐NVs, the fluorescence signal was significantly reduced after PDL1 blocking (Figure S12, Supporting Information), which indicated that PD1‐NVs could bind to hepa1‐6 cells via PD1/PDL1 interaction.

Next, we investigated the immune checkpoint blockade of PD1‐NVs by T cell‐mediated immune killing assay. Hepa1‐6 cells were incubated with PBS, pure nanovesicles (without PD1 decoration, NVs), PD1‐NVs, and anti‐PDL1 for 2 h, respectively. Then, activated CD8+T cells were added to evaluate the cytotoxic activity by using Lactate Dehydrogenase (LDH) Assay Kit. As illustrated in Figure 2D, the apoptosis rate of tumor cells pre‐treated by PD1‐NVs or anti‐PDL1 antibody significantly increased, compared to NVs group, which indicated that PD1‐NVs had an immune checkpoint blocking effect similar to anti‐PDL1 and could improve the cytotoxic activity of CD8+T cells (Figure S13, Supporting Information). Generally, IFN‐γ was secreted from activated CD8+T cells, which have anti‐tumor and immunomodulatory effects, could serve as the biomarkers of CD8+T cell activity. It was found that the content of IFN‐γ was significantly enhanced after blocking PD1/PDL1 axis by PD1‐NVs, which indicated an increasing activity of CD8+T cells (Figure S14, Supporting Information). These results demonstrated that PD1‐NVs could serve as an immune checkpoint inhibitor, to restore the activity of CD8+T cells for enhancing T cell‐mediated cytotoxic activity.

#### 2.2.3 Functional Validation of Gene Silencing

It was confirmed that CD8+ T cell‐mediated tumor immunotherapy could increase PDL1 expression in tumor cells (Figure S15, Supporting Information), which was regarded as one of the reasons for tumor immune evasion and poor response to immune checkpoint therapy. Therefore, PDL1 siRNA (siPDL1) was employed to further silence the expression of the PDL1 gene within tumor cells, aiming to enhance antitumor immunity. As shown in Figure 2E, siPDL1‐CaP and siPDL1‐CaP@PD1‐NVs both efficiently silenced PDL1 expression in hepa1‐6 cells. Additionally, siPDL1‐CaP@PD1‐NVs exhibited better gene silencing effect, it may be that the endocytosis of siPDL1‐CaP@PD1‐NVs mediated via the specific interaction between PD1‐NVs and PDL1 increases the cellular uptake of siPDL1.

#### 2.2.4 Functional Validation of Promoting the Maturation of Dendritic Cells

Dendritic cells (DCs) play an important role in initiating, regulating, and maintaining immune responses. It was reported that Ca^2+^ could act as one of the second messengers and the increased intracellular Ca^2+^ could regulate the number and proportion of immune cells in the spleen and promote the activation of DCs.^[^ ^25^ ^]^ To investigate the effect of siPDL1‐CaP@PD1‐NVs on promoting the maturation of DCs, FACS was used to analyze the expression level of CD80 and CD86 proteins on DCs. As shown in Figure 2F and Figure S16, Supporting Information, siPDL1‐CaP@PD1‐NVs showed a more significant effect (45.9 ± 0.36%) on activating DCs than that of Ca^2+^ or siPDL1‐CaP, suggesting an effective stimulus for the maturation of DCs via siPDL1‐CaP@PD1‐NVs. Therefore, the regulation of intracellular Ca^2+^ has the potential to adjust the function of immune cells, providing a new approach for tumor immunotherapy.^[^ ^26^ ^]^

#### 2.2.5 Anti‐Tumor Effect of siPDL1‐CaP@PD1‐NVs In Vitro

Given the excellent effects of siPDL1‐CaP@PD1‐NVs on immune checkpoint blockade, gene silencing, and DC activation, the cytotoxicity of T cells mediated by siPDL1‐CaP@PD1‐NVs was evaluated by the cell survival rate of tumor cells after incubating with activated T cells for 24 h, and then detecting by FACS with staining Annexin V‐FITC/PI. The cell survival rate of hepa1‐6 cells decreased down to 40.1 ± 4.03% in siPDL1‐CaP@PD1‐NVs treated group, which was lower than that of PD1‐NVs or siPDL1‐CaP treated groups (Figure 2G,H), indicating that the integration of immune checkpoint blockade (PD1‐NVs) and gene silencing of PDL1 (siPDL1‐CaP) could further enhance the cytotoxicity of T cells in vitro.

### 2.3 Anti‐Tumor Effect of siPDL1‐CaP@PD1‐NVs In Vivo

#### 2.3.1 In Vivo Distribution and Tumor Targeting Evaluation of siPDL1‐CaP@PD1‐NVs

**Figure 3:**
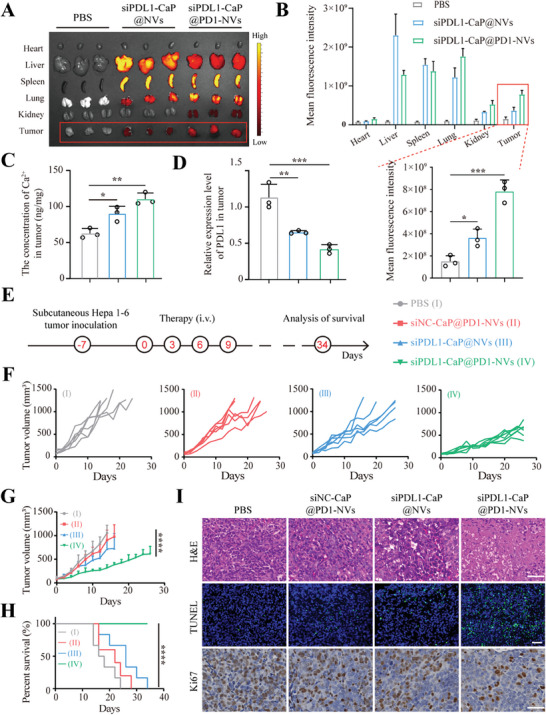
A) Ex vivo fluorescence images of mouse tumors and major organs after 48 h of tail vein injection of PBS, DiR‐labeled siPDL1‐CaP@NVs and siPDL1‐CaP@PD1‐NVs. B) The mean fluorescence intensity of mouse tumors and major organs (recorded by DiR fluorescence), *p < 0.05, **p < 0.01, ***p < 0.001, *n* = 3, one‐way ANOVA followed by Tukey's multiple comparison test. C) Ca^2+^ concentration and D) PDL1 gene expression levels of the tumor tissue (**p < 0.01, **p < 0.01, ***p < 0.001, *n* = 3, one‐way ANOVA followed by Tukey's multiple comparison test.) E) Schematic illustration of the inoculation procedure of siPDL1‐CaP@PD1‐NVs. F,G) Tumor volume changes and H) survival curve of tumor‐bearing C57BL/6 mice after receiving different treatments as indicated. ****p < 0.0001, *n* = 6, one‐way ANOVA followed by Tukey's multiple comparison test. I) Representative H&E staining, immunofluorescence staining of TUNEL and immunochemistry images of Ki‐67 after receiving different treatments as indicated. (scale bar: 40 µm).

To evaluate the delivery efficiency of siPDL1‐CaP@PD1‐NVs for tumor‐targeting in vivo, the major organs and tumor were isolated and imaged after intravenous injection of DiR labeled siPDL1‐CaP@PD1‐NVs for 48 h. As shown in **Figure** **3**A,B, siPDL1‐CaP@PD1‐NVs exhibited significantly higher fluorescent intensity in tumor than that of siPDL1‐CaP@NVs without PD1‐expressing on the cell membrane, indicating an efficient tumor targeting ability of siPDL1‐CaP@PD1‐NVs. Besides, the siRNA‐CaP@PD1‐NVs still exhibited over 30% overall retention in the blood at 8 h (Figure S17, Supporting Information), which would be helpful to enhance the delivery efficiency of siRNA drug.^[^ ^27^ ^]^

Noteworthy, we further investigated the delivery efficiency of siPDL1 and Ca^2+^ by evaluating the expression level of PDL1 and the concentration of Ca^2+^ in the aforementioned tumor, respectively. It's found that the concentration of Ca^2+^ in tumor significantly increased via the delivery of siPDL1‐CaP@PD1‐NVs (Figure 3C), meanwhile, siPDL1 loaded in siPDL1‐CaP@PD1‐NVs significantly reduced PDL1 expression in tumor (Figure 3D). These results indicated the efficient co‐delivery of siPDL1, immune checkpoint inhibitors (PD1‐NVs), and immune adjuvants (Ca^2+^) by siPDL1‐CaP@PD1‐NVs, which is of great significance in alleviating immune cell exhaustion and improving the effectiveness of immunotherapy.

#### 2.3.2 In Vivo Antitumor Effect of siPDL1‐CaP@PD1‐NVs

Inspired by the excellent effects of siPDL1‐CaP@PD1‐NVs on tumor suppression in vitro, we next established a subcutaneous liver cancer model in C57BL/6 mice to evaluate the antitumor efficacy of siPDL1‐CaP@PD1‐NVs in vivo. As displayed in Figure 3E, the Hepa1‐6‐tumor‐bearing mice after 7 days of inoculation were injected via the tail vein with PBS, siNC‐CaP@PD1‐NVs, siPDL1‐CaP@NVs and siPDL1‐CaP@PD1‐NVs, respectively. siNC‐CaP@PD1‐NVs and siPDL1‐CaP@NVs had a moderate inhibitory effect on tumor progression compared to the PBS group, which might be contributed by the immune checkpoint blockade mediated by PD1‐NVs and PDL1 gene silencing mediated by siPDL1, respectively. Whereas, the tumor progression was apparently inhibited after the administration of siPDL1‐CaP@PD1‐NVs, due to the synergistically enhanced combination effect of PD1‐NVs and siPDL1 (Figure 3F–G). Particularly, the mice treated with siPDL1‐CaP@PD1‐NVs observed a suppressed progression of tumor, while tumors in the other groups grew aggressively on the 24th day (Figure S18, Supporting Information). Over the time monitored, there was no prominent effect on the body weight of mice for siPDL1‐CaP@PD1‐NVs, indicating limited potential side‐effects on the normal physiological activity of the mice (Figure S19, Supporting Information). Additionally, siPDL1‐CaP@PD1‐NVs were also able to significantly prolong the survival of tumor‐bearing mice compared with the other groups (Figure 3H). Furthermore, we evaluate the efficacy of tumor immunotherapy of siPDL1‐CaP@PD1‐NVs via pathological diagnosis, including Ki67 staining (reflecting tumor proliferation), TUNEL staining (reflecting tumor cell apoptosis), and H&E staining (reflecting tumor cell morphology). As shown in Figure 3I, tumor cell morphology was disrupted, tumor proliferation activity was reduced and apoptosis was significantly increased in the siPDL1‐CaP@PD1‐NVs group compared to other groups. These results indicated that siPDL1‐CaP@PD1‐NVs could effectively restrain tumor progression and dramatically prolong the survival period of tumor‐bearing mice.

To further evaluate the in vivo toxicity of siPDL1‐CaP@PD1‐NVs, the serum and major organs (heart, liver, spleen, lung, kidney) were collected from the mice in different groups. H&E staining of important organs showed no obvious tissue morphological changes and intact cell morphology with distinct nucleus and cytoplasm (Figure S20, Supporting Information). Additionally, the serum biochemical markers also showed no significant change in any groups (Figure S21, Supporting Information). The results indicated that siPDL1‐CaP@PD1‐NVs possessed excellent in vivo safety with negligible side effects.

### 2.4 Remodeling the Tumor Immune Microenvironment Inducing by siPDL1‐CaP@PD1‐NVs

T‐cell exhaustion and immunosuppression restrict the efficacy of immune checkpoint blockade (ICB) therapy in HCC.^[^ ^28^ ^]^ Therefore, it's important to improve the rate of infiltration for activated T cells and regulate the immune suppressive microenvironment for the prospected application of siPDL1‐CaP@PD1‐NVs in clinical practice.

**Figure 4:**
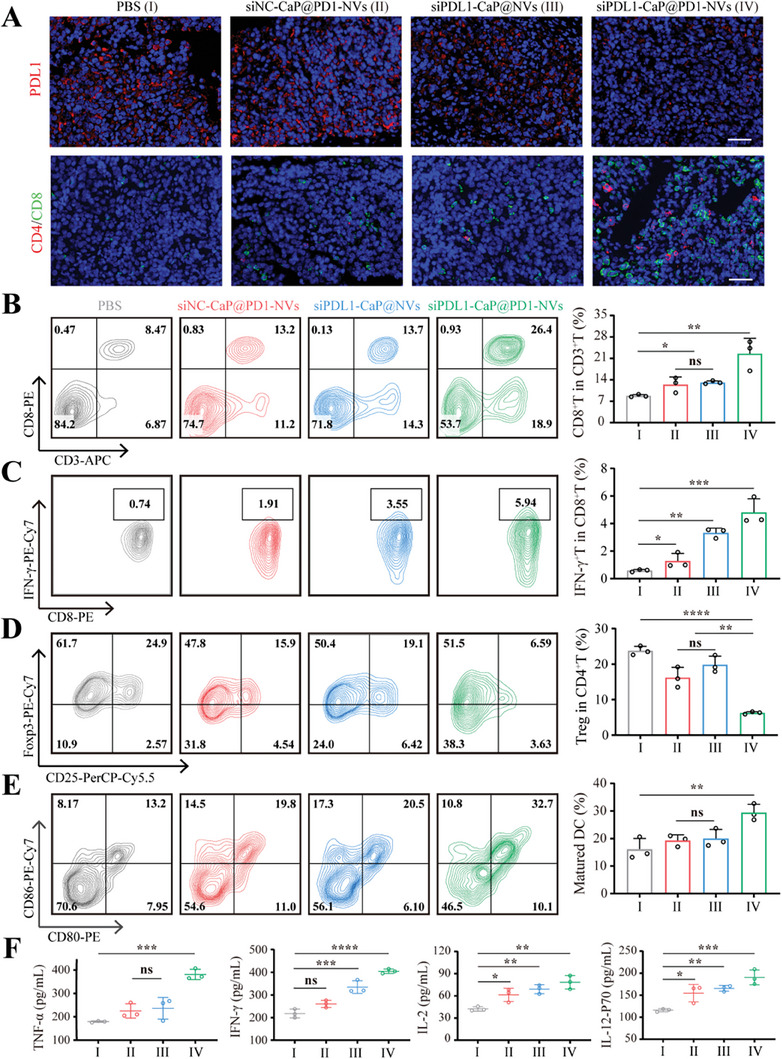
siPDL1‐CaP@PD1‐NVs reshaped the tumor immune‐suppressive microenvironment. A) Typical immunofluorescence images of PDL1 (red), CD4+T (red), and CD8+T cells (green) in tumor after receiving different treatment as indicated. (scale bar: 40 µm). FCM analysis of B–D) the ratio of CD3+CD8+ T cells, IFN‐γ‐secreting CD8+T cells, and Treg cells (CD4+ CD25+ Foxp3+ T cells) in tumor and E) the matured DCs in draining lymph nodes after receiving different treatment as indicated. (**p < 0.01, ***p < 0.001, ****p < 0.0001, *n* = 3, one‐way ANOVA followed by Tukey's multiple comparison test.) F) TNF‐α, IFN‐γ, IL‐2, and IL‐12‐P70 cytokine levels measured by Elisa analysis in tumor isolated from mice after receiving different treatment as indicated. (**p < 0.01, ***p < 0.001, ****p < 0.0001, *n* = 3, one‐way ANOVA followed by Tukey's multiple comparison test).

It's well known that PDL1, as a negative regulatory factor of the immune response, can inhibit T cell activity.^[^ ^29^ ^]^ As shown in **Figure** **4**A, siPDL1‐CaP@PD1‐NVs can significantly reduce the expression of PDL1 protein in tumor tissue, indicating a remarkable improvement in tumor immune suppression. Next, we investigate the changes in tumor‐infiltrating T cells (TILs) after receiving different treatments. The degree of CD8+ T cell infiltration (CD3+CD8+) in siPDL1‐CaP@PD1‐NVs group was significantly increased (2.7‐, 1.8‐, and 1.7‐fold, compared to PBS, siNC‐CaP@PD1‐NVs and siPDL1‐CaP@NVs group, respectively) (Figure 4B). The results were consistent with immunofluorescence analysis of tumor tissue sections, in which, the number of CD4+T cells and CD8+ T cells were significantly increased after siPDL1‐CaP@PD1‐NVs treatment. Excitingly, siPDL1‐CaP@PD1‐NVs not only induces tumor‐infiltrating CD8+T cell increase, but also further stimulates the activity of CD8+T cells, with 8.3‐, 3.8‐, and 1.4‐fold increase comparing to PBS, siNC‐CaP@PD1‐NVs and siPDL1‐CaP@NVs group, respectively (Figure 4C). In addition, the infiltration of regulatory T cells (Tregs, Foxp3+ CD25+ CD4+), which could lead to immune resistance and suppress the antitumor immune response, in siPDL1‐CaP@PD1‐NVs group was only 6.3 ± 0.34%, which was 3.8 times less than the PBS group (Figure 4D). These results indicated that siPDL1‐CaP@PD1‐NVs could effectively alleviate the immune suppressive microenvironment.

To evaluate the immune effects of siPDL1‐CaP@PD1‐NVs in vivo, considering that dendritic cells (DCs) play a crucial role in presenting tumor‐related antigens and initiating T‐cell responses in the body, we initially examined the DCs maturation in the tumor‐draining lymph nodes (LNs). The ratio of matured DC population (CD80+CD86+) in siPDL1‐CaP@PD1‐NVs group (29.4 ± 3.01%) was markedly higher than that of any other groups, indicating that siPDL1‐CaP@PD1‐NVs could effectively induce the DC maturation (Figure 4E).

Moreover, we evaluated the activation of immune cells via secretion of inflammatory cytokines in tumors after administration of siPDL1‐CaP@PD1‐NVs. TNF‐α and IFN‐γ, which have anti‐tumor and immune‐regulatory effects, increased 2.13‐ and 1.86‐fold, respectively, compared to that of PBS‐treated group. Meanwhile, IL‐2 and IL‐12‐P70, which could accelerate the proliferation and cytotoxicity of T cells and NK cells, increased 1.85‐ and 1.64‐fold, respectively (Figure 4F). These results indicated that siPDL1‐CaP@PD1‐NVs could effectively induce cytokine secretion.

These results together demonstrate that siPDL1‐CaP@PD1‐NVs not only stimulate DC maturation, promote cytokine secretion, increase the number and activity of tumor‐infiltrating CD8+ T cells, but also decrease the number of Tregs and PDL1 expression in tumor, which is beneficial for enhancing anti‐tumor immune effect and reducing immune suppression in the tumor microenvironment. Therefore, siPDL1‐CaP@PD1‐NVs can remodel the immunosuppressive microenvironment and significantly improve the efficacy of immunotherapy.

### 2.5 In Vivo Antitumor Effect of siPDL1/Pbrm1‐CaP@PD1‐NVs

Noteworthy, siPDL1‐CaP@PD1‐NVs can effectively restrain tumor progression and dramatically prolong the survival period of tumor‐bearing mice, but it was unable to completely eliminate tumor cells, which indicated the immune escape for a subset of tumor cells. It has been reported that knocking out the Pbrm1 gene can make tumor cells more sensitive to immunotherapy, reduce tumor immune escape, and improve T cell‐mediated anti‐tumor immune efficacy.^[^ ^9a^ ^]^ Therefore, Pbrm1 gene has the potential to be a target for enhancing tumor immunotherapy.

For this purpose, we fabricated siPbrm1/PDL1‐CaP@PD1‐NVs and demonstrated that it could effectively reduce the expression level of the PDL1/Pbrm1 in Hepa1‐6 cells (Figures S22 and S23, Supporting Information). To further the siPDL1‐CaP (20.2 ± 2.16%) and siPbrm1‐CaP (19.3 ± 3.61%) group, respectively, indicating the enhanced T‐cell cytotoxicity in vitro mediated by double gene silencing of PDL1 and Pbrm1 (Figure S24, Supporting Information).

**Figure 5:**
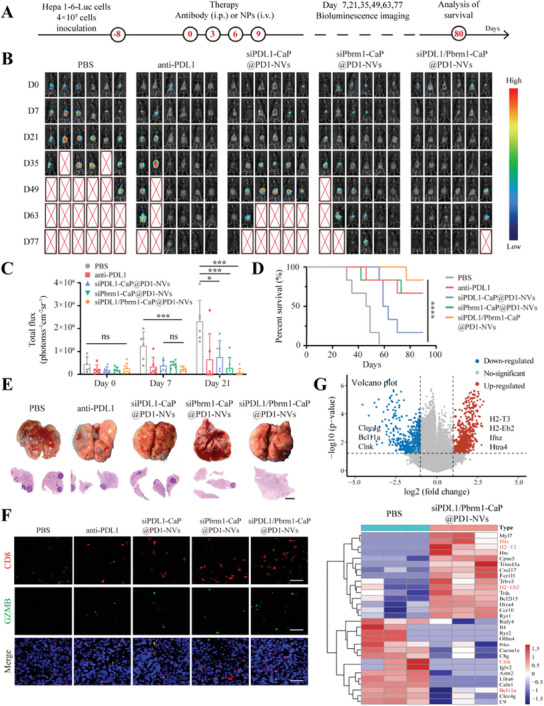
A) Schematic illustration of the inoculation procedure of orthotopic Hepa1‐6‐luc bearing mice. B) Bioluminescence imaging of orthotopic Hepa1‐6‐luc bearing mice in different groups at the 0, 7, 21, 35, 49, 63, and 77 day. C) Total flux intensity of the Hepa1‐6‐luc‐bearing mice at the 0, 7, and 21 day (ns: not significant, *p* > 0.05, *p < 0.05, ***p < 0.001, *n* = 6, one‐way ANOVA followed by Tukey's multiple comparison test.) D) The survival curve of the orthotopic tumor‐bearing mice after treatment as indicated (****p < 0.0001, *n* = 6, one‐way ANOVA followed by Tukey's multiple comparison test.) E) Photo images and H&E staining of lung and metastatic tumor isolated from mice after receiving different treatments as indicated. (scale bar, 500 µm). F) Immunofluorescence images of CD8+T cells (red) and granzyme B (GZMB, green) in tumor tissues for different treatment groups. Scale bar, 40 µm. G) Volcano plot and heat map of immune‐related genes comparing siPDL1/Pbrm1‐CaP@PD1‐NVs treated mice with PBS treated group.

To further evaluate the therapeutic efficacy of siPDL1/Pbrm1‐CaP@PD1‐NVs in vivo, the orthotopic Hepa1‐6‐luc tumor model was established, since it has more clinical similarity to actual tumors in terms of biological characteristics (**Figure** **5**A). As expected, the siPDL1‐CaP@PD1‐NVs or Pbrm1‐CaP@PD1‐NVs alone could moderately suppress the tumor progression (indicated by luciferase activity) and improve overall survival to some degree, with one out of six mice (1/6) and four out of six mice (4/6) alive at the 77th day, respectively. Inspiringly, the siPDL1/Pbrm1‐CaP@PD1‐NVs significantly inhibited the orthotopic tumor growth and prolonged the survival period, with all six mice (6/6) alive at the 77th day (Figure 5B,C). In addition, although anti‐PD‐L1 treatment represented some ability to inhibit tumor progression or extent overall survival (4/6, alive), the serous cytokines IL‐17 was significantly higher than that in siPDL1/Pbrm1‐CaP@PD1‐NVs group, indicating a higher immunogenicity. While, the serous cytokines (IL‐6 and IL‐17) and C‐reactive protein (CRP) showed no significant change compared to PBS group, indicating the low immunogenicity of siPDL1/Pbrm1‐CaP@PD1‐NVs (Figure S25, Supporting Information). It is worth noting that the surviving mice (5/6) in siPDL1/Pbrm1‐CaP@PD1‐NVs group had no detectable luciferase activity of orthotopic tumor, which indicated that the orthotopic Hepa1‐6‐luc tumor‐bearing mice might be completely cured (Figure 5D). Moreover, siPDL1/Pbrm1‐CaP@PD1‐NVs could efficiently prevent the pulmonary metastasis, whereas siPDL1‐CaP@PD1‐NVs or Pbrm1‐CaP@PD1‐NVs alone exhibited the pulmonary metastasis to some degree (Figure 5E). These findings suggested that siPDL1/Pbrm1‐CaP@PD1‐NVs had no adverse immune reactions and could more efficiently inhibit tumor growth and metastasis.

The immunosuppressive TME is the main obstacle that limits cancer immunotherapy.^[^ ^30^ ^]^ Therefore, we further examined whether siPDL1/Pbrm1‐CaP@PD1‐NVs could modulate the immunosuppressive TME. First, we found that siPDL1/Pbrm1‐CaP@PD1‐NVs could not only increase the number of tumor‐infiltrating CD8+T cells (red), but also enhance the T cell activity, as indicated by granzyme B (green), compared with that of siPDL1‐CaP@PD1‐NVs or siPbrm1‐CaP@PD1‐NVs alone (Figure 5F). Next, the RNA‐seq results demonstrated that the expression level of T cell activation‐related genes (H2‐Eb2 and H2‐T3), tumor‐killing cytokine‐related gene (Ifnz), and pro‐apoptotic regulator gene (Htra4) significantly increased after the treatment of siPDL1/Pbrm1‐CaP@PD1‐NVs. In contrast, the expression of negative regulators of natural killer cell activation gene (Clnk), T cell‐mediated immune regulation gene (Clec4g), and T cell/B cell differentiation negative regulator gene (Bcl11a) significantly decreased (Figure 5G). These findings suggested that the siPDL1/Pbrm1‐CaP@PD1‐NVs could effectively regulate the expression profiles of immune‐related genes in tumor, which contributes to enhance immune susceptibility, reduce tumor immune evasion, and improve the efficacy of tumor immunotherapy. Additionally, we also found that siPDL1/Pbrm1‐CaP@PD1‐EVs could effectively stimulate the maturation of DCs in the spleen (Figure S26, Supporting Information), which is beneficial for initiating T cell‐mediated immune responses.

To sum up, siPDL1/Pbrm1‐CaP@PD1‐NVs has the ability to extensively remodel the immunosuppressive tumor microenvironment and enhance the immune response against tumors, and the superior antitumor effect can be attributed to i) the gene silence of PDL1 and Pbrm1 to promote the immune susceptibility of tumor cells, ii) blocking immune checkpoints (PDL1) and promoting DCs maturation.

**Figure 6:**
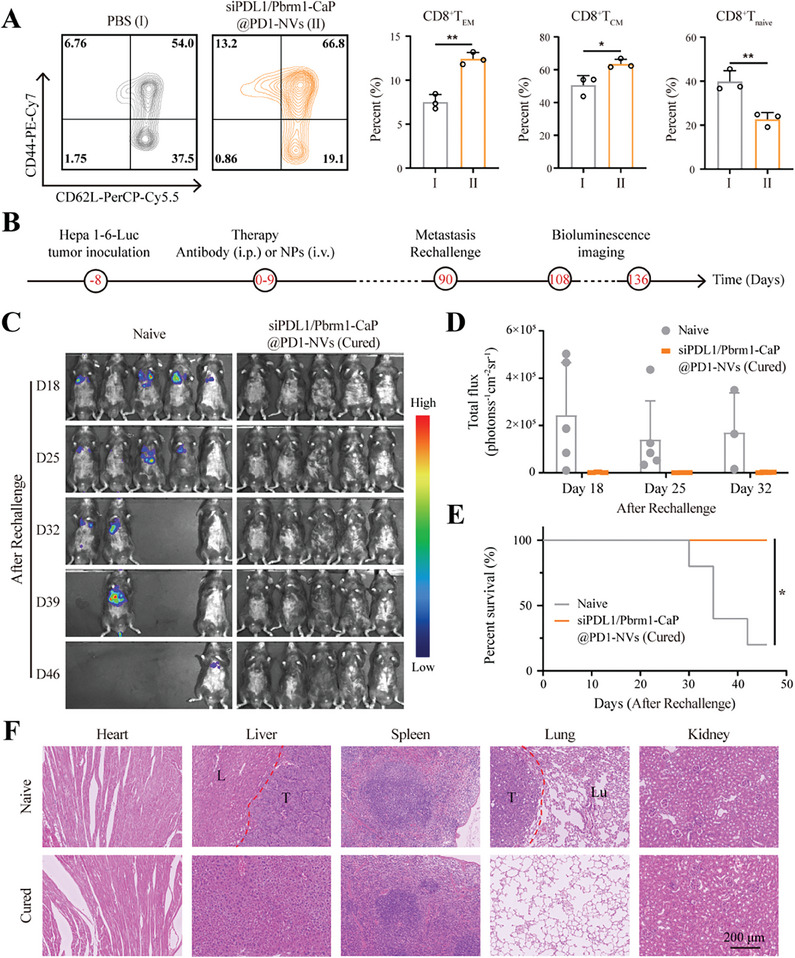
A) Flow cytometry profiles of T_EM_, T_CM_, and T_Naive_ in CD4+ T and CD8+ T cells in spleen of the orthotopic Hepa1‐6‐luc‐bearing mice in different groups at 80 days. (*p < 0.05, **p < 0.01, ****p < 0.0001, *n* = 3, two‐tailed unpaired Student's *t*‐test) B) Schematic illustration of in vivo prevention of Hepa 1–6 tumor metastasis re‐challenge in the cured mice treated with siPDL1/Pbrm1‐CaP@PD1‐NVs at the 90 day. C) Bioluminescence imaging of naive and cured mice at the 18, 25, 32, 39, and 46 day after re‐challenge as indicated. D) Total flux intensity of naive and cured mice at the 18, 25, and 32 day after re‐challenge as indicated (*n* = 5). E) The survival curve of naive and cured mice after re‐challenge as indicated. (*p < 0.05, *n* = 5, two‐tailed unpaired Student's *t*‐test.) F) H&E staining of major organs isolated from the naive and cured mice after re‐challenge as indicated. T indicated tumor tissues, L indicated liver tissues, and Lu indicated lung tissues. Scale bar, 200 µm.

To further evaluate the impact of siPDL1/Pbrm1‐CaP@PD1‐NVs on the immune memory capacity of the tumor‐bearing mice, we investigated the memory T cells in spleen by FCM. As illustrated in **Figures** **6**A and S27, Supporting Information, the siPDL1/Pbrm1‐CaP@PD1‐NVs exhibited higher frequency of CD8+T_EM_ cells (12.4 ± 0.71%) and CD8+T_CM_ cells (63.57 ± 2.82%), and lower frequency of CD8+T_Naive_ cells (22.6 ± 3.09%) in spleen, suggesting the ability of siPDL1/Pbrm1‐CaP@PD1‐NVs to generate a more potent immune memory response to prevent tumor metastasis.

Furthermore, the cured mice (*n* = 5) in the siPDL1/Pbrm1‐CaP@PD1‐NVs group were intravenously inoculated with Hepa1‐6‐luc cells (3 × 10^6^ per 100 µL) on the 90th day, and the luciferase activity (indicated tumor progression) was analyzed at the 18th day after rechallenge (Figure 6B). For the cured mice, tumor growth completely inhibited, due to the excellent ability of immune memory (Figure 6C); however, for the naive mice (same age with the cured mice), all the mice (5/5) exhibited tumor metastasis and partially died (4/5) on the 136th day (46 days after rechallenge) (Figure 6D,E). HE staining further confirmed that there was no tumor metastasis in the major organs of the cured mice (Figure 6F; Figure S28, Supporting Information). These results indicated that siPDL1/Pbrm1‐CaP@PD1‐NVs had an excellent anti‐tumor effect on primary liver cancer, not only significantly prolonged the survival period, but also prevented tumor recurrence and metastasis with a long‐term immune memory.

## 3 Conclusions

In summary, a multi‐functional biomimetic nanocarrier (siRNA‐CaP@PD1‐NVs) was developed to simultaneously and efficiently deliver nucleic acid drugs (siRNA), immune adjuvant (CaP), and quasi immune checkpoint inhibitor (PD1‐NVs). The siRNA‐CaP@PD1‐NVs displayed spherical morphology and negative charge, with excellent tumor accumulation ability and acid responsiveness, which facilitates the lysosomal escape for siRNA drug. Importantly, this multi‐functional nanocarrier could not only enhance the sensitivity of tumor cells against immunotherapy and reduce tumor immune escape via both Pbrm1/PDL1 gene silencing, but also strengthen the cytotoxic activity of immune cells by blocking PD1/PDL1 axis. Therefore, the siRNA‐CaP@PD1‐NVs improved the tumor immune‐suppressive microenvironment, enhanced the infiltration and activity of CD8+ T cells, and promoted DC cell maturation and formed long‐term immune memory, leading to significantly inhibit tumor growth or even eliminate exist tumors, and effectively prevent tumor recurrence and metastasis.

## 4 Experimental Section

### Fabrication of Genetically Engineered HEK 293FT‐PD1 Stable Cell Lines

First, the recombinant plasmid was constructed, named pCDH‐CMV‐PD1‐mCherry‐Puro, in which PD1‐mCherry fusion protein gene sequence was inserted between the XbaI cleavage site and BamHI cleavage site of pCDH‐CMV‐Puro plasmid. HEK 293FT cells were used for *Lentivirus* packaging via a Lipofectamine 3000 Transfection Kit (Thermo Fisher Scientific, USA), and the viral supernatant was collected by ultracentrifugation at 25 000 rpm (4 °C) for 90 min after incubation with HEK 293FT cells for 48 h. Then, the HEK 293FT cells were infected by aforementioned *lentivirus* and incubated with the polybrene reagent (5 µg mL^−1^, Santa Cruz biotechnology) for 48 h. Next, the HEK 293FT cells were cultured in DMEM with FBS (10%) and puromycin (5 µg mL^−1^) for 7 d, to screen the cells that stably express the PD1‐mCherry fusion protein. Finally, the genetically engineered HEK 293FT‐PD1 cells (PD1‐mCherry stable expressing) were confirmed by confocal laser microscopy (CLSM, Zeiss LSM780), flow cytometry (BD, FACSVerse, USA), and western blot.

For CLSM analysis, HEK293FT cells stably expressed the fusion protein PD1‐mCherry (red), and the cell membrane was stained by DiO (green). For flow cytometry analysis, the PD1‐expressing HEK293FT stable cells were gated by mCherry+ and the original HEK293FT cells were used as the control. For western blot analysis, the PD1‐expressing HEK293FT stable cells were lysed with RIPA buffer for 30 min on ice. The total membrane proteins were measured by BCA protein assay, after heating with the loading buffer to 100 °C for 10 min, the samples were loaded onto 10% SDS‐PAGE, operating at 100 V for 1 h. Then, the samples were incubated with PD‐1 primary antibodies (1 µg mL^−1^, abcam, ab214421) overnight at 4 °C after blocking membranes with 5% BSA for 1.5 h, followed by the corresponding secondary antibodies (1/2000, abcam, ab288151) for 1.5 h at 25 °C. Finally, it was analyzed by immunoblotting analysis (recorded by the ChemiDoc MP imaging system).

### Synthesis and Characterization of siRNA‐CaP@PD1‐NVs

The siRNA‐CaP@PD1‐NVs were synthesized via a modified protocol.^[^ ^12a^ ^]^ For the calcium‐loaded microemulsion, 40 µL CaCl_2_ (0.5 m, pH = 7) and 10 µL siRNA (100 µm) were added into the oil phase containing Igepal‐520 and cyclohexane (3:7, v/v). The sequence of siRNA is listed in Table S1 (Supporting Information). For the phosphate buffer‐loaded microemulsion, 74 µL Na_2_HPO_4_ solution (0.1 m, pH = 9) and 74 µL DOPA (35 mm) were added into the oil phase containing Igepal‐520 and cyclohexane (3:7, v/v). The separate microemulsions (3 mL each) were stirred for 10 min at 25 °C, then mixed and stirred for 60 min to generate the inner core of siRNA‐CaP. Afterwards, an equal volume of 100% EtOH was added for demulsification, and the mixture was centrifuged at 12 000 g (4 °C) for 15 min. The siRNA‐CaP was collected and washed with 100% EtOH to eliminate any remaining organic solvents. Next, the siRNA‐CaP was suspended in 800 µL chloroform, and mixed with 30 µL DOPC (35 mM) and 10 µL DOTAP (35 mm), then the mixture was transferred into 1 mL ddH_2_O and subjected to a cell ultrasound breaker in an ice bath for 5 min (on for 2 s, off for 3 s). Finally, the siRNA‐CaP NPs could be obtained by removing the excess chloroform in a rotary evaporator at 35 °C and 100 rpm for 3 min. For synthesis of siRNA‐CaP@PD1‐NVs, the above‐mentioned PD1 expressing cell membrane and siRNA‐CaP NPs were gently mixed (mass ratio = 1:1, based on the weight of total membrane proteins) and then successively passed through 800 nm and 400 nm pore‐sized polycarbonate (PC) filters for 10 times. For synthesis of PD1‐NVs, the above‐mentioned PD1 expressing cell membrane was successively passed through 800 nm and 400 nm pore‐sized PC filters for 10 times.

The morphology of siRNA‐CaP and siRNA‐CaP@PD1‐NVs was observed with transmission electron microscopy (TEM, FEI Company, Hillsboro, OR). The hydrated particle size and surface charge property (zeta potential) were determined by dynamic light scatterings (DLS, Malvern Zetasizer), and the average size was monitored for 72 h in PBS buffer (pH = 7.4) to evaluate the stability of siRNA‐CaP@PD1‐NVs. The UV‐vis absorbance of siRNA, PD1‐NVs, siRNA‐CaP, siRNA‐CaP@PD1‐NVs was detected by a microplate reader (Spectra Max M5, Germany), and the fluorescence image of siRNA, PD1‐NVs, siRNA‐CaP, siRNA‐CaP@PD1‐NVs (siRNA: GGAAAAGGAAGAUGAGCAATT‐Alexa Fluor 555, excited by 561 nm) was acquired by using the ChemiDoc MP imaging system.

To calculate the loading efficiency of siRNA, the siRNA (labeled by FAM) was employed to fabricate siRNA‐CaP@PD1‐NVs, then it was degraded to release siRNA. The siRNA content was calculated by the fluorescence intensity of the released siRNA and the standard curve.

The specific calculation formula is as follows:

(1) $$\begin{array}{ccc} \mathrm{Loading}\;\mathrm{efficiency}\left(\%\right) & = & \left(\mathrm{Loaded}\;\mathrm{amount}\;\mathrm{of}\;\mathrm{drug}/\mathrm{Feeding}\;\mathrm{amount}\;\mathrm{of}\;\mathrm{drug}\right)\\ & & \times \;100\% \end{array}$$

To verify the successful coating on siRNA‐CaP NPs with the PD1 expressing cell membrane, the protein profiles of siRNA‐CaP, PD1‐NVs, and siRNA‐CaP@PD1‐NVs were determined by Coomassie Brilliant Blue staining (Sigma‐Aldrich, USA). To confirm the orientation of PD1 proteins on siRNA‐CaP@PD1‐NVs, an immunoprecipitation (IP) assay was conducted. In brief, the siRNA‐CaP@PD1‐NVs were dispersed in PBS buffer (1 mL), and then mixed with 50% protein A/G agarose beads (100 µL) at 4 °C with gentle rotation for 2 h. Afterwards, the mixture was incubated overnight at 4 °C with PD1 primary antibody (10 µg mL^−1^, abcam) or IgG (as a control). Finally, the beads were gently washed 3 times with PBS buffer, and the samples were evaluated by western blot.

To investigate the acid response release ability of siRNA‐CaP@PD1‐NVs, the siRNA‐CaP@PD1‐NVs were resuspended in PBS buffer (pH 5.5 or 7.4). The supernatant (0.5 mL) was withdrawn at the predetermined time intervals, to assess the released amount of calcium ion (Ca^2+^) using the calcium colorimetric assay kit after centrifugation. Moreover, TEM was employed to the morphology of siRNA‐CaP@PD1‐NVs after incubation in PBS (pH 5.5 or 7.4) for 24 h.

### In Vitro Anti‐Tumor Effect of siPDL1‐CaP@PD1‐NVs

Hepa 1–6 cells (1.5 × 10^5^) were seeded in a 24‐well plate and cultured with PBS, PD1‐NVs, siPDL1‐CaP (20 µg mL^−1^) and siPDL1‐CaP@PD1‐NVs (20 µg mL^−1^) for 2 h. Next, the activated CTLs (CD8+T cells) were added into Hepa 1–6 cells (CTLs:Hepa 1–6 = 1:1, number ratio) for 24 h. Finally, the cells were stained by using Annexin V‐FITC/PI (DOJINDO Laboratories, NX610) and anti‐CD3‐APC antibody (eBioscience, 12‐0081‐82) and detected by FCM (BD, FACSVerse, USA).

### Animals

C57BL mice (male, 6–8 weeks, 20–22 g) were purchased from Shanghai Slack Laboratory Animal Co., LTD and were cared according to the guidelines stated in the Guide for the Care and Use of Laboratory Animals. All the experiments are approved by the Animal Ethics Committee of Mengchao Hepatobiliary Hospital of Fujian Medical University (MCHH‐AEC‐2022‐03).

### In Vivo Anti‐Tumor Efficacy of Subcutaneous Tumor Model

To evaluate the anti‐tumor effects of siPDL1‐CaP@PD1‐NVs, the C57BL mice (6‐8 weeks) were s.c. injection of Hepa 1–6 cells (3 × 10^6^). When the tumor size reached 50–100 mm^3^, the Hepa 1‐6‐bearing mice were randomly divided into 4 groups (*n* = 6), and injected with PBS, siNC‐CaP@PD1‐NVs, siPDL1‐CaP@NVs, and siPDL1‐CaP@PD1‐NVs via tail‐vein for a total of 4 times in every 3 days. Injection dose: 687.5 µg per time, measured by membrane protein content; siPDL1 was 55 µg kg^−1^. The tumors size was measured by using vernier calipers every other day from day 0 to day 34, and the tumor size (V) was calculated according to V = a × b^2^/2, in which, *a* is the longest diameter and *b* is the shortest diameter of the tumor. The body weight was monitored to assess potential toxicities at the same time points. Considering the animal ethics, when the mice exhibited marked features of impaired health or when the tumor size exceeded 1200 cm^3^, the mice were sacrificed. The tumor tissues were collected and fixed by 4% paraformaldehyde, and dehydrated in a gradient concentration of alcohol solution sequentially. After embedded in paraffin, the tumor tissues were sectioned and the slices were stained with hematoxylin‐Eosin (H&E), Ki67 (ab15580, Abcam Company, USA), and TUNEL (Servicebio, China), according to the manufacturer's instructions.

To evaluate the immune responses, the Hepa 1–6 tumor‐bearing mice (*n* = 3) in different groups were sacrificed at the 3rd day after the last administration. The tumors and drainage lymph nodes were collected to prepare the cell suspension according to the previously reported method. Briefly, the tumors or drainage lymph nodes were digested using collagenase type IV (1 mg mL^−1^), hyaluronidase (0.2 mg mL^−1^), and DNase I (0.02 mg mL^−1^) at 37 °C for 2 h. The single‐cell suspension was obtained after being filtered using a cell‐strainer. To evaluate the DC maturity, DC cells from drainage lymph nodes were collected, and detected by FCM (staining with anti‐CD11c‐APC, anti‐CD80‐PE, and anti‐CD86‐PE‐Cy7, respectively). Moreover, to investigate the activation of TILs, the TILs from tumor were harvested and detected by FCM, specifically, CD8+T cells were stained by anti‐CD3‐APC and anti‐CD8‐PE; the activated CD8+T cells were stained by anti‐CD3‐APC, anti‐CD8‐PE and anti‐INF‐γ‐PE‐Cy7; Tregs were stained by anti‐CD3‐APC, anti‐CD4‐FITC, anti‐CD25‐PerCP‐Cy5.5, and anti‐Foxp3‐PE‐Cy7.

Besides, to assess the cytokines in tumors, the aforementioned tumors were collected and lysed in RIPA lysis buffer including 1% phenylmethylsulfonyl fluoride (PMSF) and 2% cocktail protease inhibitor, and then grinned with grinding beads by the tissue crusher at low temperature for 6 min. Afterward, the suspension was centrifuged at 14 000 g (4 °C) for 5 min. Finally, the supernatants were measured by ELISA Kit (Boster Biological Technology, USA) to detect the TNF‐α, IFNγ, IL‐2, and IL‐12p70 according to the standard protocols.

Furthermore, for the immunofluorescence analysis of CD4/CD8+ T cells and PDL1 protein in tumor, the aforementioned tumors were harvested and soaked in 4% paraformaldehyde and fixed for 24 h, followed by paraffin embedding and section, and immunofluorescence staining according to standard protocols.

### RNA Sequencing and Bioinformatic Analysis

The orthotopic hepa1‐6‐bearing mice (*n* = 3) received siPDL1/Pbrm1‐CaP@PD1‐NVs or PBS were sacrificed on the 3^rd^ day after the last dose. The tumor tissues were isolated, and mRNA was extracted according to the aforementioned method. RNA was then subjected to RNA library preparation using VAHTS Stranded mRNA‐seq Library Prep Kit. Next, RNA‐seq was conducted on Illumina Navoseq 6000 system. The sequence data was processed according to the standard transcriptome sequencing pipeline. The differentially expressed genes were identified using R package limma provided by Bioconductor (www.bioconductor.org) and heatmap of selected genes related to immune were drawn using pheatmap via R software (version 3.6.3).

### Statistical Analysis

All the data are presented as the mean ± SD (*n* ≥ 3). One‐way ANOVA or student's *t*‐test was performed on different experimental data by the GraphPad Prism 8 software, and the significance was marked by asterisks (ns: not significant, *p* > 0.05, *p < 0.05, **p < 0.01, ***p < 0.001, ****p < 0.0001). Survival curves were generated using Kaplan‐Meier estimates and tested using the log‐rank test.

## Conflict of Interest

The authors declare no conflict of interest.

## Author Contributions

Y.S. and Y.L. contributed equally to this work. Y.S. performed conceptualization, data curation, formal analysis, investigation, writing of original draft, and project administration. Y.L. performed investigation. R.L. performed investigation. C.Z. performed formal analysis. M.W. performed formal analysis. X.Z. performed formal analysis. A.Z. performed formal analysis. N.L. performed formal analysis. Y.Z. performed formal analysis. H.X. performed formal analysis. R.Z. performed formal analysis. Y.Z. performed supervision, and funding acquisition. X.L. performed supervision, conceptualization, funding acquisition, and editing. All authors read and approved the final version of the submitted manuscript.

## Supporting information

Supporting Information

## Data Availability

The data that support the findings of this study are available from the corresponding author upon reasonable request.

## References

[1] a) D. S. Chen , I. Mellman , Immunity 2013, 39, 1; 23890059 10.1016/j.immuni.2013.07.012

[2] a) A. H. Sharpe , K. E. Pauken , Nat. Rev. Immunol. 2018, 18, 153; 28990585 10.1038/nri.2017.108

[3] a) M. Wu , H. Li , C. Zhang , Y. Wang , C. Zhang , Y. Zhang , A. Zhong , D. Zhang , X. Liu , Adv. Sci. 2023, 10, 2206399; 10.1002/advs.202206399 PMC10131848 36840638

[4] W. Zeng , M. Yu , T. Chen , Y. Liu , Y. Yi , C. Huang , J. Tang , H. Li , M. Ou , T. Wang , M. Wu , L. Mei , Adv. Sci. 2022, 9, 2201703. 10.1002/advs.202201703 PMC9376744 35678111

[5] Y. X. Lin , Y. Wang , J. Ding , A. Jiang , J. Wang , M. Yu , S. Blake , S. Liu , C. J. Bieberich , O. C. Farokhzad , L. Mei , H. Wang , J. Shi , Sci. Transl. Med. 2021, 13, eaba9772. 34162754 10.1126/scitranslmed.aba9772 PMC8284983

[6] a) G. P. Dunn , A. T. Bruce , H. Ikeda , L. J. Old , R. D. Schreiber , Nat. Immunol. 2002, 3, 991; 12407406 10.1038/ni1102-991

[7] a) K. Yamamoto , A. Venida , J. Yano , D. E. Biancur , M. Kakiuchi , S. Gupta , A. S. W. Sohn , S. Mukhopadhyay , E. Y. Lin , S. J. Parker , R. S. Banh , J. A. Paulo , K. W. Wen , J. Debnath , G. E. Kim , J. D. Mancias , D. T. Fearon , R. M. Perera , A. C. Kimmelman , Nature 2020, 581, 100; 32376951 10.1038/s41586-020-2229-5 PMC7296553

[8] D. Miao , C. A. Margolis , W. Gao , M. H. Voss , W. Li , D. J. Martini , C. Norton , D. Bossé , S. M. Wankowicz , D. Cullen , C. Horak , M. Wind‐Rotolo , A. Tracy , M. Giannakis , F. S. Hodi , C. G. Drake , M. W. Ball , M. E. Allaf , A. Snyder , M. D. Hellmann , T. Ho , R. J. Motzer , S. Signoretti , W. G. Kaelin , T. K. Choueiri , E. M. Van Allen , Science 2018, 359, 801. 29301960 10.1126/science.aan5951 PMC6035749

[9] a) D. Pan , A. Kobayashi , P. Jiang , L. Ferrari de Andrade , R. E. Tay , A. M. Luoma , D. Tsoucas , X. Qiu , K. Lim , P. Rao , H. W. Long , G. C. Yuan , J. Doench , M. Brown , X. S. Liu , K. W. Wucherpfennig , Science 2018, 359, 770; 29301958 10.1126/science.aao1710 PMC5953516

[10] a) R. S. Riley , C. H. June , R. Langer , M. J. Mitchell , Nat. Rev. Drug Discovery 2019, 18, 175; 30622344 10.1038/s41573-018-0006-z PMC6410566

[11] a) W. Yu , C. Xuan , B. Liu , L. Zhou , N. Yin , E. Gong , Z. Zhang , Y. Li , K. Zhang , J. Shi , Nano Res. 2023, 16, 735;

[12] a) K. W. Huang , F. F. Hsu , J. T. Qiu , G. J. Chern , Y. A. Lee , C. C. Chang , Y. T. Huang , Y. C. Sung , C. C. Chiang , R. L. Huang , C. C. Lin , T. K. Dinh , H. C. Huang , Y. C. Shih , D. Alson , C. Y. Lin , Y. C. Lin , P. C. Chang , S. Y. Lin , Y. Chen , Sci. Adv. 2020, 6, eaax5032; 31998834 10.1126/sciadv.aax5032 PMC6962042

[13] a) C. H. Liu , G. J. Chern , F. F. Hsu , K. W. Huang , Y. C. Sung , H. C. Huang , J. T. Qiu , S. K. Wang , C. C. Lin , C. H. Wu , H. C. Wu , J. Y. Liu , Y. Chen , Hepatology 2018, 67, 899; 28885731 10.1002/hep.29513

[14] a) Y. Li , S. Gong , W. Pan , Y. Chen , B. Liu , N. Li , B. Tang , Chem. Sci. 2020, 11, 7429; 34123024 10.1039/d0sc00293c PMC8159290

[15] Y. Wang , Q. Zhao , B. Zhao , Y. Zheng , Q. Zhuang , N. Liao , P. Wang , Z. Cai , D. Zhang , Y. Zeng , X. Liu , Adv. Sci. 2022, 9, 2105631. 10.1002/advs.202105631 PMC9009112 35142445

[16] a) L. Zhu , X. Yu , T. Cao , H. Deng , X. Tang , Q. Lin , Q. Zhou , Acta Pharm. Sin. B 2023, 13, 2464; 37425034 10.1016/j.apsb.2023.03.004 PMC10326251

[17] X. Ma , L. Kuang , Y. Yin , L. Tang , Y. Zhang , Q. Fan , B. Wang , Z. Dong , W. Wang , T. Yin , Y. Wang , ACS Nano 2023, 17, 2341. 36688797 10.1021/acsnano.2c09033

[18] a) Y. Yu , Q. Cheng , X. Ji , H. Chen , W. Zeng , X. Zeng , Y. Zhao , L. Mei , Sci. Adv. 2022, 8, eadd3599; 36490349 10.1126/sciadv.add3599 PMC9733928

[19] a) L. Ding , X. Zhang , P. Yu , F. Peng , Y. Sun , Y. Wu , Z. Luo , H. Li , Y. Zeng , M. Wu , X. Liu , Mol. Ther. 2023, 31, 2489; 37087570 10.1016/j.ymthe.2023.04.011 PMC10422002

[20] Y. Huang , M. Chen , Y. Shen , X. Shen , M. Li , Y. Li , Y. Liu , K. Cai , Z. Luo , Y. Hu , Chem. Eng. J. 2023, 470, 144145.

[21] R. Kanasty , J. R. Dorkin , A. Vegas , D. Anderson , Nat. Mater. 2013, 12, 967. 24150415 10.1038/nmat3765

[22] a) A. Rajeev , A. Siby , M. J. Koottungal , J. George , F. John , ChemistrySelect 2021, 6, 13350;

[23] Z. Dong , L. Feng , Y. Hao , Q. Li , M. Chen , Z. Yang , H. Zhao , Z. Liu , Chem 2020, 6, 1391.

[24] Y. Sun , L. Mo , X. Hu , D. Yu , S. Xie , J. Li , Z. Zhao , X. Fang , M. Ye , L. Qiu , W. Tan , Y. Yang , ACS Nano 2022, 16, 21129. 36484532 10.1021/acsnano.2c09093

[25] a) Y. Y. Lin , X. Wang , X. Huang , J. Zhang , N. Xia , Q. Zhao , Expert Rev. Vaccines 2017, 16, 895.; 28712326 10.1080/14760584.2017.1355733

[26] M. Trebak , J. P. Kinet , Nat. Rev. Immunol. 2019, 19, 154. 30622345 10.1038/s41577-018-0110-7 PMC6788797

[27] a) Y. Zhai , J. Wang , T. Lang , Y. Kong , R. Rong , Y. Cai , W. Ran , F. Xiong , C. Zheng , Y. Wang , Y. Yu , H. H. Zhu , P. Zhang , Y. Li , Nat. Nanotechnol. 2021, 16, 1271; 34580467 10.1038/s41565-021-00972-7

[28] S. C. Wei , C. R. Duffy , J. P. Allison , Cancer Discov 2018, 8, 1069. 30115704 10.1158/2159-8290.CD-18-0367

[29] H. Zhang , Z. Dai , W. Wu , Z. Wang , N. Zhang , L. Zhang , W. J. Zeng , Z. Liu , Q. Cheng , J. Exp. Clin. Canc. Res. 2021, 40, 184. 10.1186/s13046-021-01987-7 PMC8178863 34088360

[30] H. Phuengkham , L. Ren , I. W. Shin , Y. T. Lim , Adv. Mater. 2019, 31, 1803322. 10.1002/adma.201803322 30773696

