# Supplementary material for: Multifunctional Biomimetic Nanocarriers for Dual‐Targeted Immuno‐Gene Therapy Against Hepatocellular Carcinoma
Source: Adv Sci (Weinh). 2024 Jul 8;11(34):2400951. doi: 10.1002/advs.202400951 (PMC11425963; doi:10.1002/advs.202400951)
Supplement: Supplementary file 1 — Supporting Information [file ADVS-11-2400951-s001.docx]

**Multifunctional Biomimetic Nanocarriers for Dual-targeted Immuno-gene Therapy Against Hepatocellular Carcinoma**

*Yupeng Sun,^#[a,b]^ Yan Liu,^#[a,c]^ Rui Li,^[a,c]^ Cuilin Zhang,^[a,b]^ Ming Wu,^[a,b]^ Xiaolong Zhang,^[a,b]^ Aixian Zheng,^[a,b]^ Naishun Liao,^[a]^ Youshi Zheng,^[a]^ Haipo Xu,^[a]^ Rui Zeng,^[a]^ Yongyi Zeng,*^[a,d]^ and Xiaolong Liu*^[a,b,e]^*

1. The United Innovation of Mengchao Hepatobiliary Technology Key Laboratory of Fujian Province

Mengchao Hepatobiliary Hospital of Fujian Medical University

Fuzhou 350025, P. R. China

E-mail: [xiaoloong.liu@gmail.com;](mailto:xiaoloong.liu@gmail.com;) [lamp197311@126.com](mailto:lamp197311@126.com).

1. Mengchao Med-X Center

Fuzhou University

Fuzhou 350116, P. R. China

1. College of Biological Science and Engineering

Fuzhou University

Fuzhou, 350116, PR China

1. Liver Disease Center

The First Affiliated Hospital of Fujian Medical University

Fuzhou 350005, P. R. China

1. CAS Key Laboratory of Design and Assembly of Functional Nanostructures,

Fujian Institute of Research on the Structure of Matter, Chinese Academy of Sciences

Fuzhou 350002, P. R. China

DOI:

**Materials and Methods**

**Materials**. Calcium chloride (CaCl_2_) and disodium hydrogen phosphate (Na_2_HPO_4_) were obtained from Sinopharm Chemical Reagent (Sinopharm Chemical Reagent Co., Ltd., China). 1,2-dioleoyl-sn-glycero-3-phosphate (sodium salt) (DOPA), 1,2-dioleoyl-sn-glycero-3-phosphocholine (DOPC) and 1,2-dioleoyl-3-trimethylammonium-propane (chloride salt) (DOTAP) were purchased from Ruixi Biotechnology (Ruixi Biotechnology Co., Ltd., China). The PD-L1 antibody (anti-PD-L1), Pbrm1 antibody (anti-Pbrm1) and PD1 antibody (anti-PD1) were purchased from Abcam (ABCM, USA). Dulbecco’s modified Eagle’s medium (DMEM), foetal bovine serum (FBS), penicillin and streptomycin, trypsin-ethylenediaminetetraacetic acid (EDTA) were purchased from Thermo Fisher Scientific (Thermo Fisher Scientific, Inc., USA). Cell Counting Kit (CCK-8) and Live/Dead staining Kit were purchased from Dojindo Laboratories (Kumamoto, Japan). DiI and DiO were purchased from Beyotime Biotechnology (Shanghai, China). Mouse adenovirus antibody (IgG) ELISA Kit (CSB-E13901m) was purchased from CUSABIO Biotech CO., Ltd. (Wuhan, China). All of the antibodies for flow cytometric analysis were purchased from eBioscience (eBioscience, USA). Analytical grades of all other chemicals were obtained from Sinopharm Chemical Reagent Co. Ltd.

**Isolation of cell membrane over-expressing PD1**. The cell membrane over-expressing PD1 was isolated from HEK293FT-PD1 cells (stably expressing PD1 protein). In brief, HEK293FT-PD1 cells were harvested with a scraper and re-suspended at a concentration of 5 × 10^7^cells /ml in ice-cold membrane protein extraction reagent A (Biyuntien, P0033) including 1% phenylmethylsulfonyl fluoride (PMSF) and 2% Cocktail protease inhibitor. After resting on ice for 30 min, the cells were quickly frozen in liquid nitrogen, and then melted at room temperature, and frozen and thawed repeatedly three times to disrupt the cells. The homogenate of cells was centrifuged at 7000 g (4℃) for 10 min, then the supernatant was collected and centrifuged at 100000 g (4℃) for 60 min. Finally, the cell membrane was re-suspended with PBS (0.01M, pH 7.4) to obtain cell membrane suspension (total membrane proteins, 10mg/mL). The total membrane proteins were detected by BCA protein assay, and the PD1 protein content in the cell membrane was determined by mouse PD1 ELISA kit.

**Cell culture**. Hepa1-6 cells (mouse liver cancer), Hepa1-6-luc cells (stably expressing Luciferase), Hepa1-6-PDL1-EGFP cells (stably expressing PDL1-EGFP protein) and HEK293FT-PD1 cells (stably expressing PD1 protein) were maintained in DMEM medium with FBS (10%) and penicillin-streptomycin (100 U mL^-1^). All the cells were grown at 37 ℃, 5% CO_2_, and 95% air humidity.

***In vitro* cytotoxicity assay**. To assess the cytotoxicity of siRNA-CaP@PD1-NVs, Hepa1-6 cells were cultured in 96-well plate with 1.5×10^4^ cells per well and incubated for 24 h. Then, the cells were cultured by the fresh medium with different concentrations of siRNA-CaP NPs or siRNA-CaP@PD1-NVs (calculated by Ca^2+^, 0, 5, 10, 20, 30, and 40 µg mL^-1^) for 24 h. Afterward, the cell viability was measured via a CCK-8 Kit according to the previously reported works.[^2^](#_ENREF_2)

**Cellular Uptake and lysosome escape of siRNA-CaP@PD1-NVs.** The cellular uptake of siRNA-CaP@PD1-NVs was investigated by flow cytometry (BD, FACSVerse, USA). Briefly, Hepa1-6 cells were cultured in 6-well plate for 24 h at 37℃ with 3×10^5^ cells per well. Then, the fresh medium with Alexa Fluor^TM^ 555 labeled siRNA-CaP@PD1-NVs (20 μg mL^-1^) was added into the aforesaid Hepa 1-6 cells, and cultured for 4 h, 8 h, 12 h, or 24 h. Next, the Hepa 1-6 cells were collected and suspended in 0.2 mL PBS buffer, subsequently analyzed by flow cytometry (gated by Alexa Fluor^TM^ 555).

The lysosome escape of siRNA-CaP@PD1-NVs was evaluated by confocal microscopy (LSM 780, Germany). Similarly, 1.5×10^5^ Hepa 1-6 cells were inoculated in a confocal dish for 24 h at 37℃. Then, the fresh medium with Alexa Fluor^TM^ 555 labeled siRNA-CaP@PD1-NVs (20 μg mL^-1^) was added into the aforesaid Hepa 1-6 cells, and cultured for another 4 h or 8 h. Next, the treated Hepa 1-6 cells were washed twice by PBS buffer, and stained with 1×Hoechst 33342 dye and LysoTracker^TM^ Green DND-26 for 15 min, subsequently observed by CLSM after a gentle PBS wash. The Pearson`s correlation coefficient was calculated by ImageJ version 1.46r software, according to previous reports (AJP: Cell Physiology, 2011, 300(4):C723-C742).

To investigate the formation of autophagosomes during cellular uptake, LC3B protein was used as the autophagosome marker. Specifically, siRNA-CaP@PD1-NVs (20 μg mL^-1^) was added into the Hepa 1-6 cells (1.5×10^5^), and cultured for another 2h, 4h, 8h, 12h and 24h. Next, the Hepa 1-6 cells were lysed with RIPA buffer for 30 min on ice. The total proteins were measured by BCA protein assay, after heating with the loading buffer to 100℃ for 10 min, the samples were loaded onto 10% SDS-PAGE, operating at 100 V for 1 h. Then, the samples were incubated with anti-LC3B (1 μg/mL, abcam, ab192890) overnight at 4 ℃ after blocking with 5% BSA for 1.5 h, followed by the corresponding secondary antibodies (1/2000, abcam, ab288151) for 1.5 h at 25℃. Finally, it was analyzed by immunoblotting analysis (recorded by the ChemiDoc^TM^ MP imaging system).

**Isolation of CD8+ T cells.** CD8+T cells were isolated from mouse spleen according to the previously reported method (Advanced Science, 2023, 10, 2206399). Briefly, the spleen was collected from C57BL/6 mice (6-8 weeks) and immersed in PBS buffer. Firstly, the cells were flushed out from spleen with PBS buffer via a syringe. Then, the cell suspension was filtered with a 40 μm cell-strainer to eliminate the large debris. Afterwards, the cells were centrifuged at 800×g for 5 min, and re-suspended in the red blood cell lysis buffer for 5 min at 25℃, followed by centrifugation for 5 min at 800×g. Next, the CD8+ T cells were isolated by CD8+ T Cell Isolation Kit (Miltenyi Biotec) according to the included protocol. The CD8+T cells were incubated in a 6-well plate with the KBM 581 medium containing IL-2 (10 ng mL^-1^, R&D systems, MX2918061), anti-CD3ε (5 μg mL^-1^, biolegend, 100331) and anti-CD28 (2 μg mL^-1^, biolegend, 102112) to activate the CD8+T cells. It's worth noting that the CD8+T cells were expanded in vitro with the KBM 581 medium only containing IL-2 (the anti-CD3ε and anti-CD28 antibody were only added at the first time for activation).

**Function identification of siRNA-CaP@PD1-NVs as immune checkpoint blockade.** To prove the specific binding between PD1-NV_S_ and the PDL1 proteins on the surface of tumor cells, Hepa 1-6 cells (1×10^5^) were cultured in the confocal dishes overnight, then PD1-NV_S_ labeled with DiI (a dye for cell membranes) were added into the medium and incubated for 60 min at 4 ℃. Afterwards, the cells were washed with PBS, fixed with 4% paraformaldehyde for 15 min, and stained with DAPI for 15 min before observed by CLSM (Zeiss LSM 780, Germany). Meanwhile, the control group was pre-treated with anti-PDL1 (10 μg mL^-1^) for 2 h before the PD1-NV_S_ were added. Furthermore, a co-localization assay of PD1 from PD1-NV_S_ and PDL1 from Hepa 1-6 cells was performed. In brief, the plasmid (pCDH-CMV-PDL1-EGFP-Puro) was instantaneously transferred into Hepa 1-6 cells for 24 h by Lipofectamine® 3000 kit. Then, the transient transfected Hepa 1-6 cells (1.5×10^5^) were cultured in a confocal dish overnight and followed by the incubation with PD1-NV_S_ (50 μg mL^-1^ based on total membrane proteins) for 60 min at 4 ℃. Finally, the fluorescence co-localization imaging was performed by CLSM as mentioned above.

To demonstrate the immune checkpoint blockade of PD1-NV_S_, a CTL cytotoxicity assay was carried out. Briefly, Hepa 1-6 cells (3×10^5^) in 6-well plate were cultured with free NVs (50 μg mL^-1^ based on total membrane proteins), PD1-NV_S_ (50 μg mL^-1^ based on total membrane proteins), anti-PDL1 antibody (10 μg mL^-1^) or equal PBS for 2 h, then the activated CTLs (CD8+T cells) were added into Hepa 1-6 cells (CTLs:Hepa 1-6 = 1:1, number ratio). After incubating for 24 h, the culture medium was collected for determining the apoptosis rate of Hepa 1-6 cells via a Lacate Dehydrogenase (LDH) Assay Kit (Solarbio, BC0680-50) and the IFNγ via an IFNγ ELISA Kit (Boster, EK1115), according to the operation instruction.

**Real-Time Quantitative PCR (RT-qPCR) Analysis.** To prove the function of siPDL1-CaP@PD1-NVs for silencing PDL1 gene, 2×10^6^ Hepa 1-6 cells were incubated in 6-well plate with siPDL1-CaP@PD1-NVs (20 μg mL^-1^), siPDL1-CaP (20 μg mL^-1^) or PBS for 8 h, afterwards, the fresh medium was used to culture the aforementioned Hepa 1-6 cells for another 24 h. Similarly, the validation of Pbrm1 gene silence in Hepa 1-6 cells was carried out through the same protocol.

Total RNA was extracted by a TransZol Up Plus RNA Kit according to the instructed protocol. Next, the reverse transcription was performed by Hifair Ⅱ Strand cDNA Synthesis Kit. In brief, a total volume of 10 μL of mixture containing 2 μL 5× gDNA digester buffer, 1 μL gDNA digester, 500 ng of RNA and RNase-free water was incubated at 42 ℃ for 2 min. Then, 2 μL 5× Hifair II buffer plus, 2 μL Hifair II enzyme mix, 2 μL 50 μM Oligo (dT)_18_ and RNase-free water were added into the above reaction mixture with the final volume of 20 μL. At last, the reverse transcription program was performed at 25 ℃ for 5 min, 42 ℃ for 30 min, 85 ℃ for 5 min. The produced cDNA samples were then stored at -20 °C for future use.

A typical qPCR reaction mix contains 10 μL SYBR Select master mix (2×), 2 μL cDNA, 1 μL Forward Primer (10 μM), 1 μL Reverse Primer (10 μM) and 6 μL RNase-free water. RT-qPCR analysis was performed on a Bio-Rad CFX96. The relative gene expression was calculated by the 2^−ΔΔCt^ method, referring to the expression of β-actin mRNA. The primers for RT-qPCR are listed in Table S2.

To further verify the capability of siPbrm1/PDL1-CaP@PD1-NVs to decrease the expression level of the PDL1/Pbrm1, we have carried out western blot (WB) analysis. Specially, Hepa 1-6 cells (2×10^6^) were incubated in 6-well plate with siPbrm1/PDL1-CaP@PD1-NVs (20 μg mL^-1^) or PBS for 8 h, afterwards, the fresh medium was used to culture the aforementioned Hepa 1-6 cells for another 48 h. For western blot analysis, the operation process is basically the same as above, specifically,, the samples were incubated with PD-L1 primary antibodies (1/1000, abcam, ab213480) or Pbrm1 primary antibodies (1/10000, abcam, ab196022) overnight at 4 ℃ after blocking membranes with 5% BSA for 1.5 h, followed by the corresponding secondary antibodies (1/2000, abcam, ab288151) for 1.5 h at 25℃.

**Isolation of DC cells from mouse bone marrow.** The isolation of DC cells was similar to the aforesaid protocol of CD8+ T cell isolation. Firstly, the femur and tibia from C57BL/6 mice (6-8 weeks) were transferred to a fresh and cold RPMI 1640 medium, the bone was cut off via a scissor to expose the bone marrow cavity, and followed by rinsing repeatedly with the medium via a syringe. The subsequent red blood cell lysis and purification followed the aforesaid protocol of CD8+ T cells isolation. Finally, the cells were cultured in RPMI 1640 medium containing mouse granulocyte/macrophage colony stimulating factor (mGM-CSF, 20 ng mL^-1^, R&D systems, 415-ML-020/CF) and IL-4 (10 ng mL^-1^, 404-ML-010/CF) to induce DC differentiation for further usage.

**DC maturation.** To demonstrate the function of siPDL1-CaP@PD1-NVs for promoting DC maturation, the immature DC cells were extracted from mouse bone marrow and incubated with PBS, Ca^2+^ (CaCl_2_ solution), siPDL1-CaP (20 μg mL^-1^) and siPDL1-CaP@PD1-NVs (20 μg mL^-1^) for 48 h. Then, the DC cells were collected and stained with the antibody of CD11c-APC (eBioscience™, 17-0114-82), CD80-PE (eBioscience™, 12-0801-82) and CD86-PE-Cy7 (eBioscience™, 25-0862-82). At last, the DC cells were washed with PBS for 3 times, re-dispersed with 300 mL PBS buffer, and detected by flow cytometry (BD, FACSVerse, USA).

**In vivo distribution of** **siRNA-CaP@PD1-NVs.** To explore the targeting ability of siRNA-CaP@PD1-NVs, the C57BL mice (6-8 weeks) were subcutaneously (s.c.) injected with Hepa 1-6 cells (3 × 10^6^ cells) in PBS solution. As the tumor size reached approximately 100 mm^3^, the tumor-bearing mice were administered with DiR labeled siRNA-CaP@NVs or siRNA-CaP@PD1-NVs by tail-vein injection. After 48 h, the tumors and major organs were collected and imaged by an UniNano NIR-II imaging system.

For intra-tumoral Ca^2+^ concentration detection, 20 mg of the aforementioned tumor was clipped and added with 200 mL lysate, and then grinned with two grinding beads by the tissue crusher at low temperature for 6 min. Afterward, the suspension was collected by centrifuging at 14000 g (4℃) for 5 min, and measured by a Calcium Ion Detection Kit. For intra-tumoral PDL1 gene expression, 50 mg of the aforementioned tumor was clipped and added with 1 mL Tranzol Up, and then grinned with two grinding beads by the tissue crusher at low temperature for 6 min. The total RNA extraction and RT-qPCR were performed by the above-mentioned protocol.

For in vivo pharmacokinetics, we administered siRNA-CaP@PD1-NVs (siRNA was labeled by ICG) to C57BL/6 mice intravenously (IV, tail vein), the blood circulation times were assessed by the fluorescence intensity of ICG in blood after IV administration at 30 min, 2 h, 4 h, 8 h, 12 h, 24 h and 48 h post injection. And the fluorescence intensity of ICG in blood was determined by an UniNano NIR-II imaging system.

**In vivo biocompatibility of siRNA-CaP@PD1-NVs.** The C57BL mice (6-8 weeks) were injected with PBS, siNC-CaP@PD1-NVs, siPDL1-CaP@NVs and siPDL1-CaP@PD1-NVs by tail-vein injection (n = 5), respectively. 14 days after injection, for serum biochemical indicator and blood routine test, the blood was collected from the orbital sinus of mice, and the serum samples were obtained by centrifuging at 3000 g for 5 min. For hematoxylin-Eosin (H&E) pathological staining, the visceral organs (heart, liver, spleen, lungs and kidneys) were soaked in 4% paraformaldehyde and fixed for 24 h, followed by paraffin embedding and section, and H&E staining according to standard protocols.

**In vivo anti-tumor efficacy of orthotopic tumor model.** To investigate the synergistic anti-tumor effects of siPDL1/Pbrm1-CaP@PD1-NVs, hepa1-6-Luc cells (4×10^5^) were mixed with matrigel (25 μL) and then injected into the liver lobe of C57BL mice (6-8 weeks) to establish the orthotopic hepa1-6-bearing mice model. At the 8^th^ day after tumor implantation, the bioluminescence signals (indicated tumor size) were acquired by an UniNano imaging system after injection of luciferin via the tail vein (15 mg mL^-1^, 200 µL per mouse). Afterward, the hepa1-6-bearing mice were randomly assigned to receive PBS, anti-PDL1, siPDL1-CaP@PD1-NVs, siPbrm1-CaP@PD1-NVs and siPDL1/Pbrm1-CaP@PD1-NVs with i.v. injection for a total of 4 times in every 3 days, respectively. The tumor progression was monitored by bioluminescence imaging every 7 days starting from day 0 to day 77.

Finally, on the 80^th^ day, the hepa 1-6 tumor-bearing mice (n=3) were sacrificed. For the immunofluorescence analysis of CD8+ T cells and Granzyme B protein in tumor, the tumor tissues were harvested and soaked in 4% paraformaldehyde and fixed for 24 h, followed by paraffin embedding and section, and immunofluorescence staining according to the aforementioned protocols. Moreover, to investigate the lung metastasis of the orthotopic hepa1-6-bearing mice, the lung tissues were collected from treated mice to photograph, and fixed with 4% paraformaldehyde, then dehydrated in a gradient concentration of alcohol solution sequentially. After embedded in paraffin, the tissues were sectioned for H&E staining. In addition, to investigate the DC maturity, DC cells were isolated from spleen and detected by FCM (staining by anti-CD11c-APC, anti-CD80-PE and anti-CD86-PE-Cy7, respectively). For the evaluation of immune memory effect, on the 80^th^ day, the spleen was extracted and prepared a cell suspension. The cells were stained by anti-CD3-APC, anti-CD4-FITC, anti-CD8-PE, anti-CD44-PE-Cy7, anti-CD62L-PerCP-Cy5.5 and detected by FCM.

For the re-challenge study, the cured C57BL mice from the above-mentioned siPDL1/Pbrm1-CaP@PD1-NVs group (n=5) were injected with 3×10^6^ hepa1-6-Luc cells via tail-vein; while, the healthy mice (Naive) with the same age were selected as the control. Subsequently, bioluminescence imaging was used to monitor the tumor size and the survival time was also recorded. To further evaluate tumor metastasis, on the 46^th^ day after re-challenge, the major organs were isolated and fixed with 4% paraformaldehyde, followed by slicing and staining with H&E.

To further evaluate the immune response in Hepa1-6-luc-bearing mice, we established the orthotopic Hepa1-6-luc tumor model as described above. The hepa1-6-bearing mice were randomly assigned to receive PBS, anti-PDL1 and siPDL1/Pbrm1-CaP@PD1-NVs, for the serous cytokines (IL-6, IL-17) and the serous C-reactive protein (CRP) test, the blood was collected from the orbital sinus of mice 7 days after injection, and the serum samples were obtained by centrifuging at 3000 g for 5 min. The serum was measured by ELISA Kit (Boster Biological Technology, USA) to detect the IL-6 and IL-17 according to the standard protocols.

**Supplementary Figures**

**
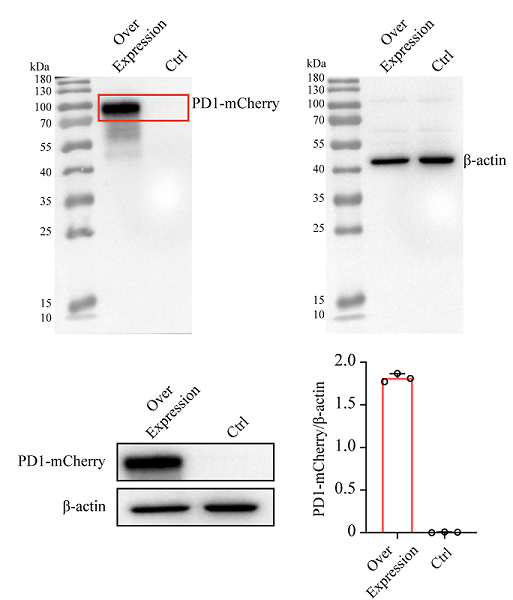
**

**Figure S1**. Western blot assay of the expression of PD1 protein in genetically engineered HEK 293FT-PD1 (Over Expression) and HEK293FT (Ctrl) cells. β-actin was used as the loading control.

**
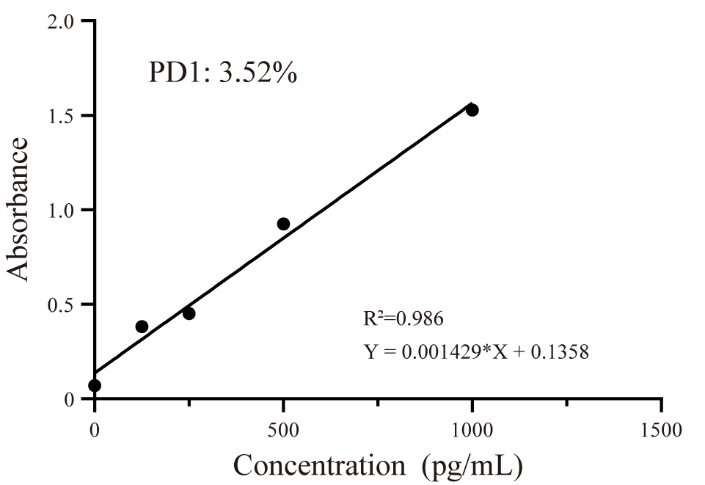
**

**Figure S2**. The PD1 content in the PD1-expressing cell membrane isolated from the genetically engineered HEK 293FT-PD1 cells. Data are presented as mean ± S.D (n = 3).


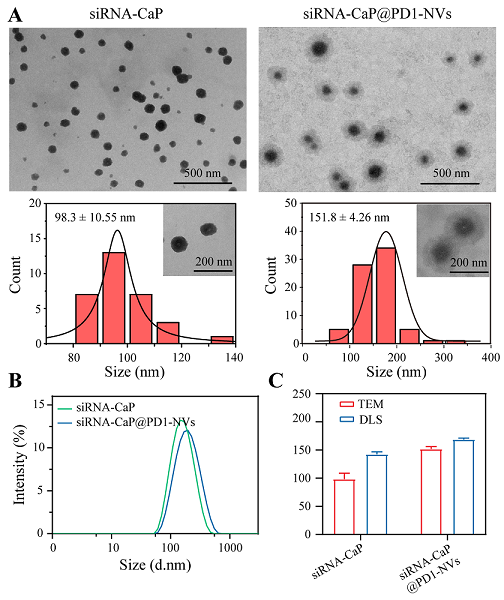


**Figure S3**. (A) Representative TEM images and the size distribution of siRNA-CaP and siRNA-CaP@PD1-NVs. (B) Hydrodynamic size distribution of siRNA-CaP and siRNA-CaP@PD1-NVs measured by DLS. (C) The particle sizes analysis of siRNA-CaP and siRNA-CaP@PD1-NVs were measured by TEM or DLS.


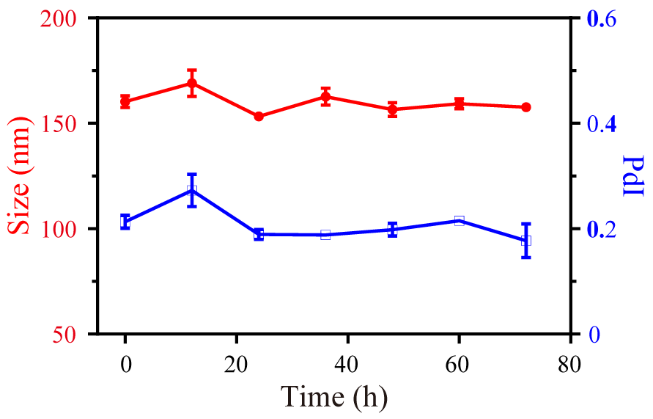


**Figure S4**. Hydrodynamic size and PDI of siRNA-CaP@PD1-NVs under physiological conditions (pH 7.4) for 72h. Data are presented as mean ± S.D (n = 3).


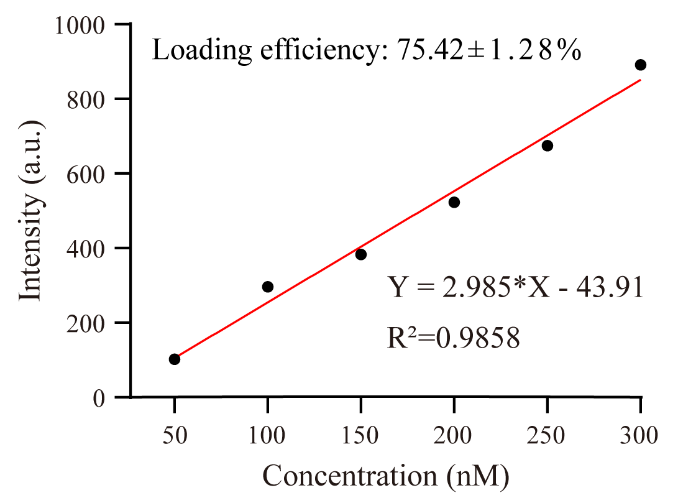


**Figure S5**. The loading efficiency of siRNA in siRNA-CaP@PD1-NVs. Data are presented as mean ± S.D (n = 3).


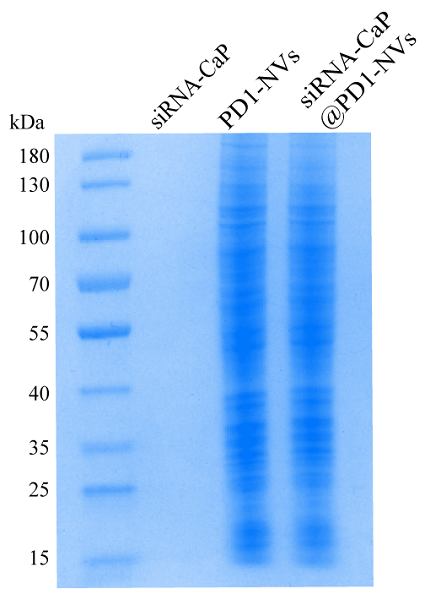


**Figure S6**. The protein profile analysis of siRNA-CaP, PD1-NVs, and siRNA-CaP@PD1-NVs by SDS-PAGE (Coomassie blue staining).


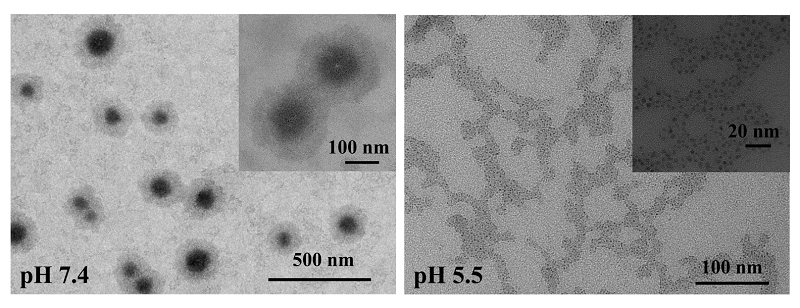


**Figure S7**. Representative TEM images of siRNA-CaP@PD1-NVs incubated at different pH values (5.5 and 7.4) for 24 hours.


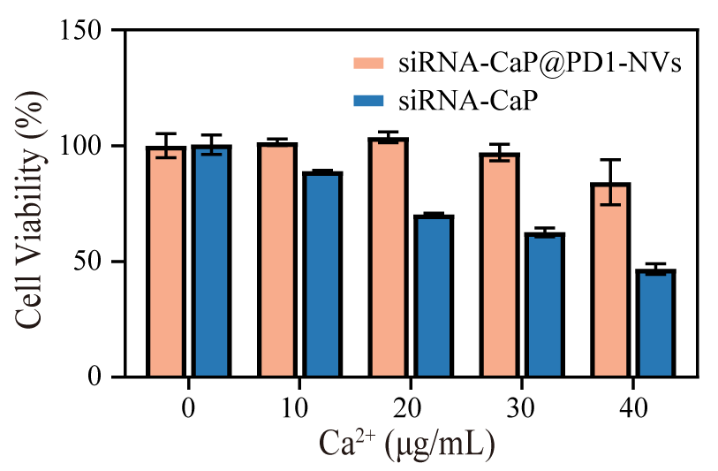


**Figure S8**. The Cell viability of Hepa 1-6 cells treated with various doses of siRNA-CaP and siRNA-CaP@PD1-NVs after 24 hours incubation, respectively. Data are presented as mean ± S.D (n = 4).


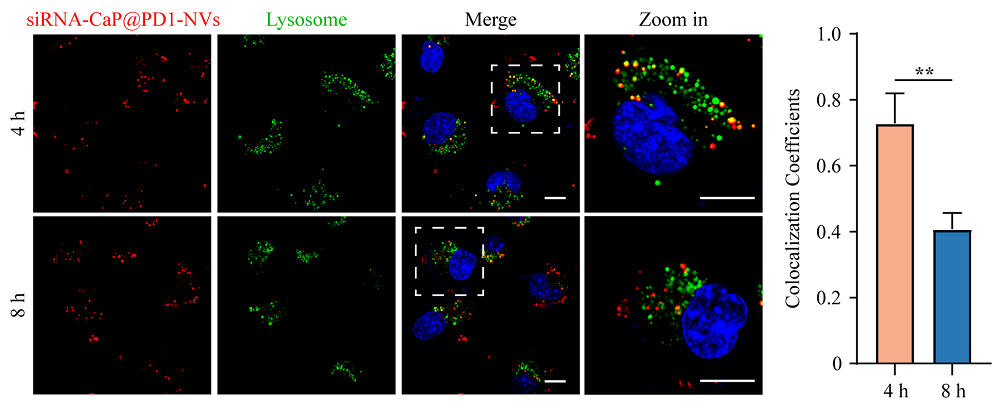


**Figure S9**. Confocal images of lysosomal escape of siRNA-CaP@PD1-EVs and co-localization statistics after incubation for 4 and 8 h in Hepa 1-6 cells, scale bar: 10 μm. The siRNAs (red) were labelled by Alexa Fluor^TM^ 555, nucleus (blue) were stained with Hoechst 33342 and lysosome (green) were indicated by LysoTracker Green.


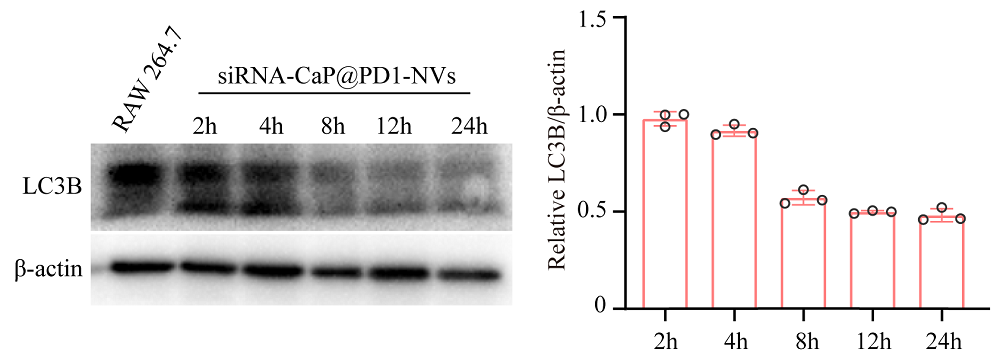


**Figure S10**. Western blot assay of the formation of autophagosomes in Hepa 1-6 cells during cellular uptake of siRNA-CaP@PD1-NVs. Lane 1: RAW 264.7 cell lysate (positive control); Lane 2 - Lane 6: 2h, 4h, 8h, 12h, 24h after the addition of the siRNA-CaP@PD1-NVs. β-actin was used as the control.


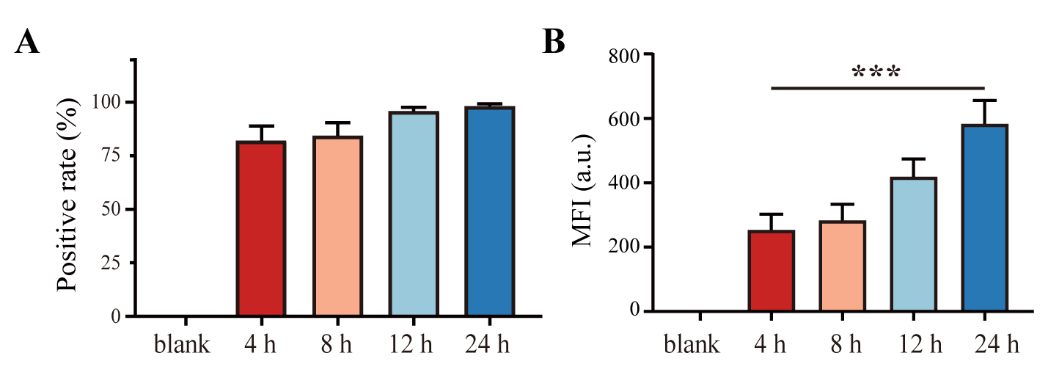


**Figure S11**. (A) The cell positivity and (B) mean fluorescence intensity of Hepa1-6 cells treated with siRNA-CaP@PD1-NVs for different incubation times. Data are presented as mean ± S.D (n = 3). ***p < 0.001.


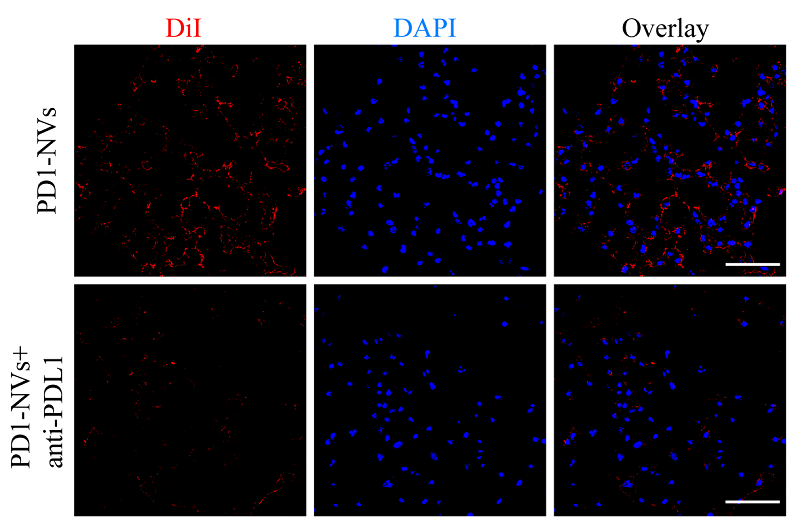


**Figure S12**. Fluorescent images of DiI-labeled PD1-NVs after co-incubation with Hepa 1-6 cells or anti-PDL1 antibody pre-treated Hepa 1-6 cells for 2 h (scale bar:100 μm).


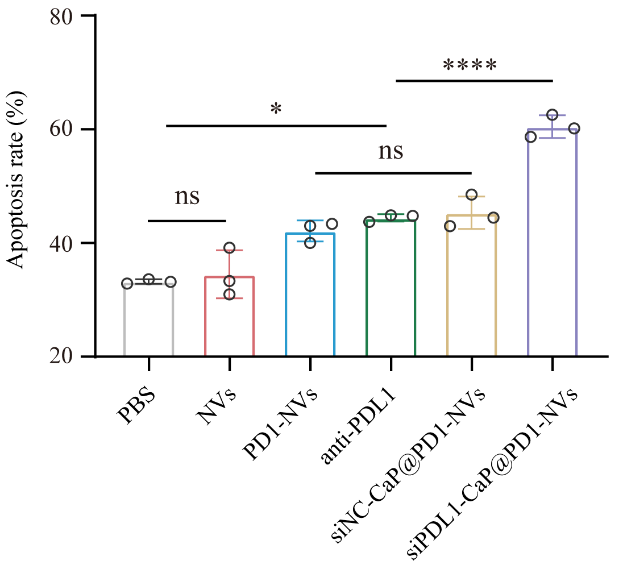


**Figure S13**. Apoptosis rate of Hepa 1-6 cells treated with PBS, pure nanovesicles (NVs), PD1-NVs, PDL1 antibody (anti-PDL1), siNC-CaP@PD1-NVs or siPDL1-CaP@PD1-NVs after co-incubation with activated CD8+T cells for 24 h detected by LDH assay.

**
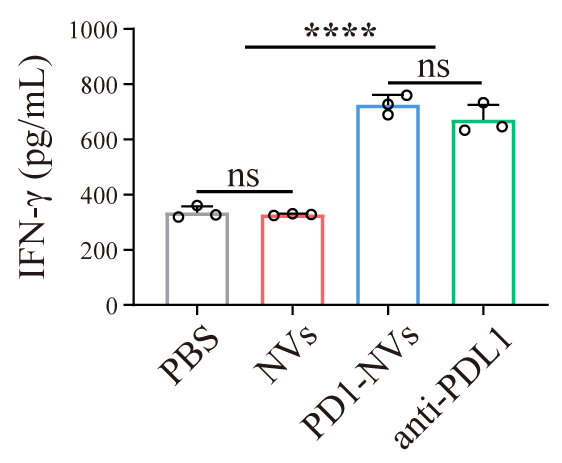
**

**Figure S14**. The concentration of IFN-γ after treatment with PBS, free NVs (NVs), PD1-NVs or PDL1 antibody (anti-PDL1) for 24 h, respectively. (*p < 0.05, **p < 0.01, ****p < 0.0001, n=3).


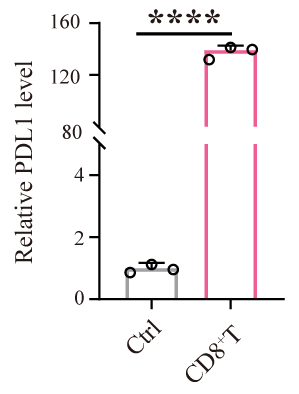


**Figure S15**. PDL1 gene expression in Hepa 1-6 cells after co-incubation with CD8+ T cells for 24 h. (****p < 0.0001, n=3)


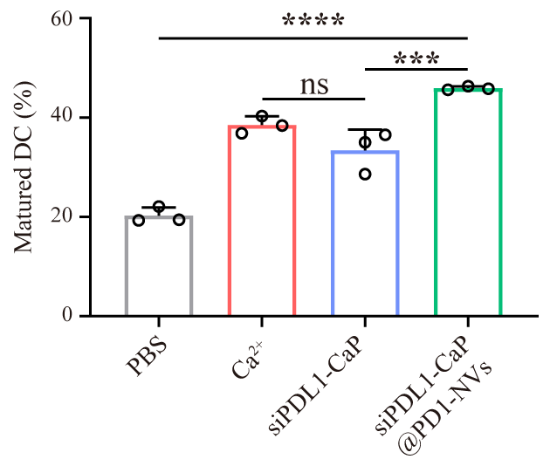


**Figure S16**. The maturation of DC cells after co-incubation with PBS, Ca^2+^, siRNA-CaP, and siRNA-CaP@PD1-NVs for 48 h, respectively. (***p < 0.001, ****p < 0.0001，n=3).


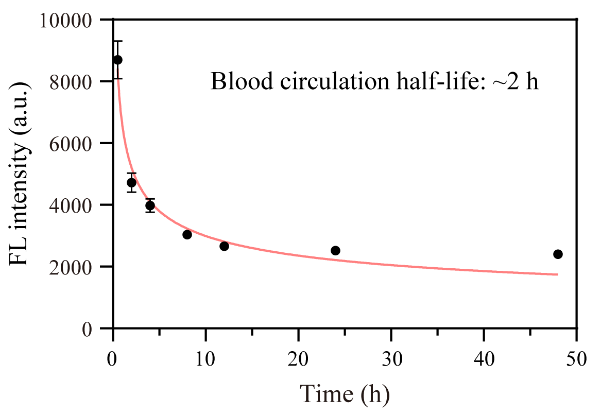


**Figure S17**. The fluorescence intensity of ICG in blood at 30 min, 2 h, 4 h, 8 h, 12 h, 24 h and 48 h after tail intravenous injection of siRNA-CaP@PD1-NVs (siRNA was labeled by ICG). Data are presented as mean ± SD, n = 3.


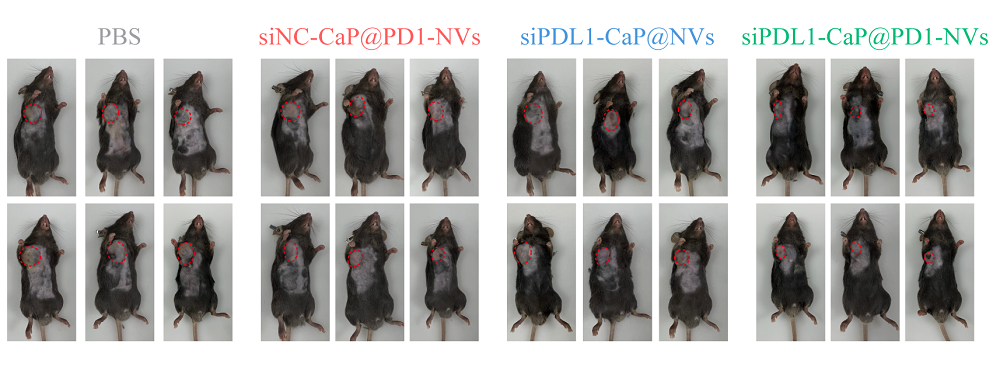


**Figure S18**. Photographs of Hepa1-6 tumor-bearing C57BL/6 mice after receiving different treatment as indicated at the end of the treatment cycle (red circles mark tumor locations).


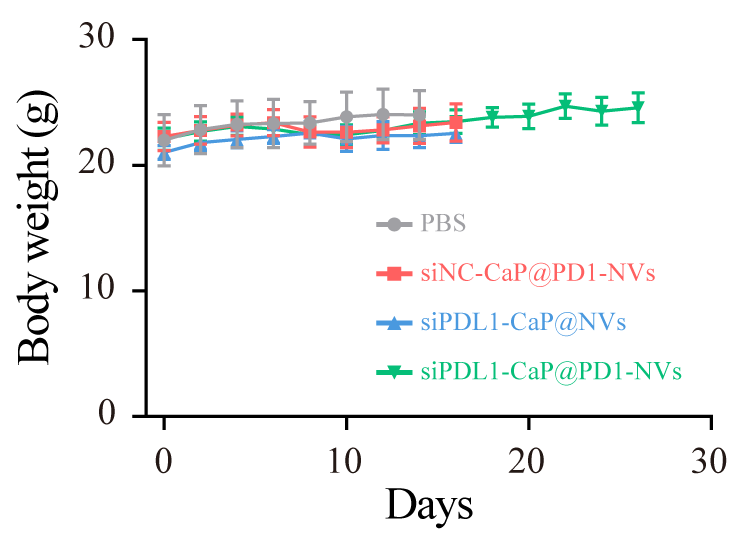


**Figure S19**. The body weight of Hepa1-6 tumor-bearing C57BL/6 mice after receiving different treatment as indicated. (****p < 0.0001, n=6).


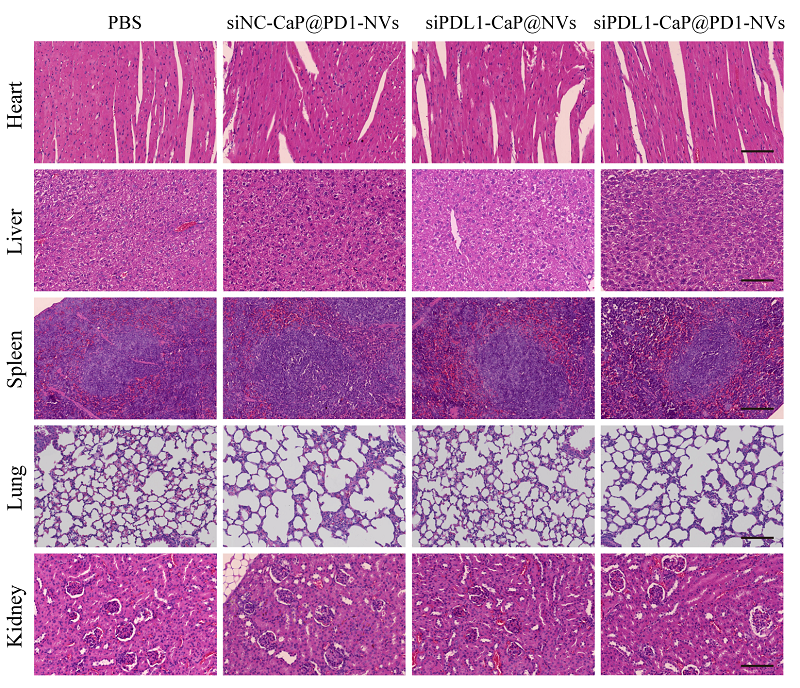


**Figure S20**. H&E staining of major organs of C57BL/6 mice after receiving different treatment as indicated. (scale bar: 100 μm).


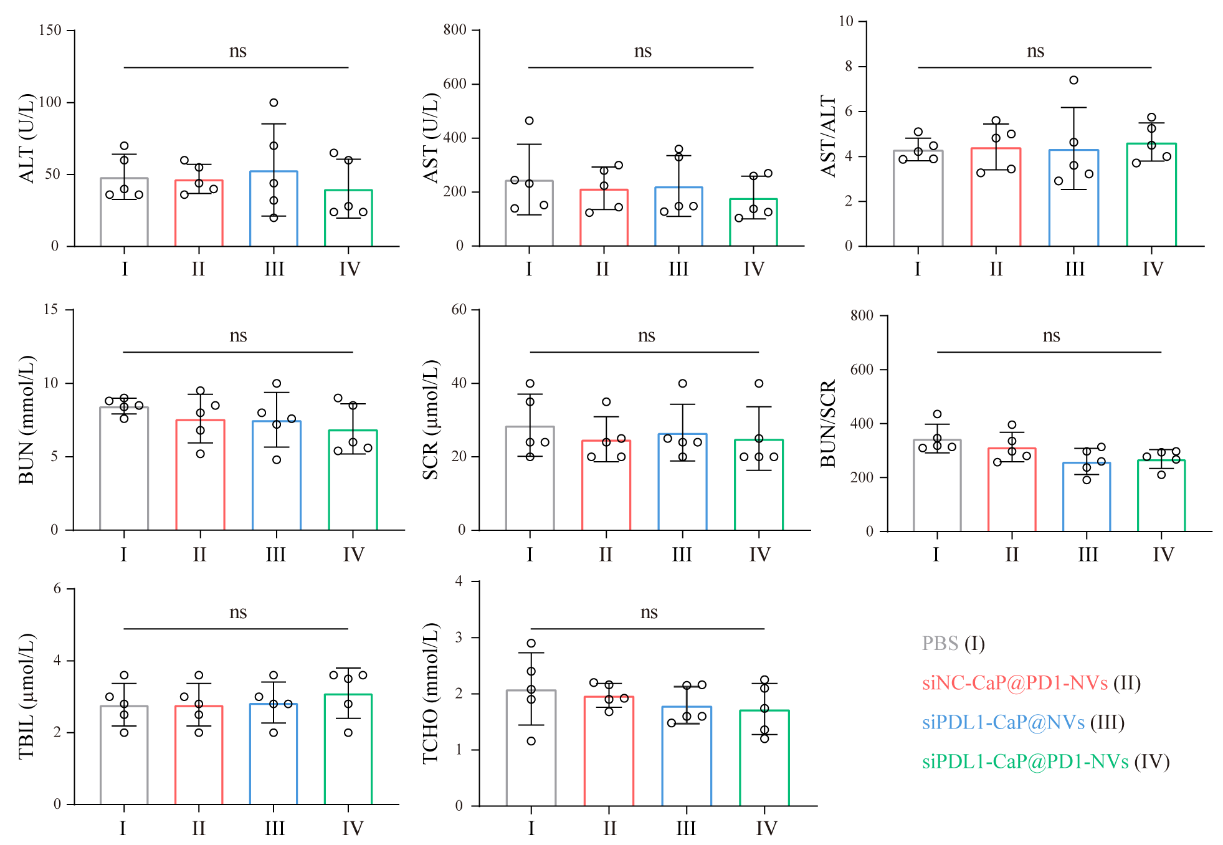


**Figure S21**. Serum biochemical indicator and blood routine test of mice after indicated treatments. ns represents not significant, Data are presented as mean ± S.D. (n = 5).


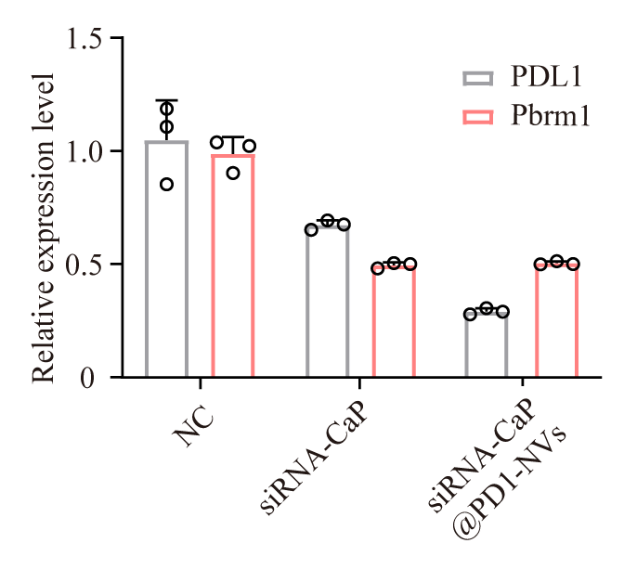


**Figure S22**. PDL1/Pbrm1 gene expression in Hepa 1-6 cells incubated with PBS (NC), siPbrm1-CaP, and siPbrm1-CaP@PD1-NVs for 24 h, respectively.


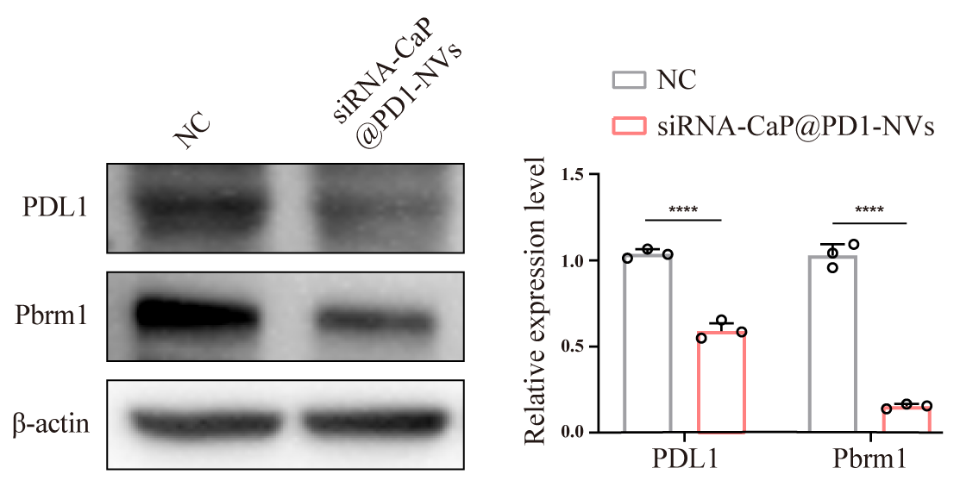


**Figure S23**. Western blot assay of the expression of PDL1 and Pbrm1 protein in Hepa1-6 cells. β-actin was used as the loading control.


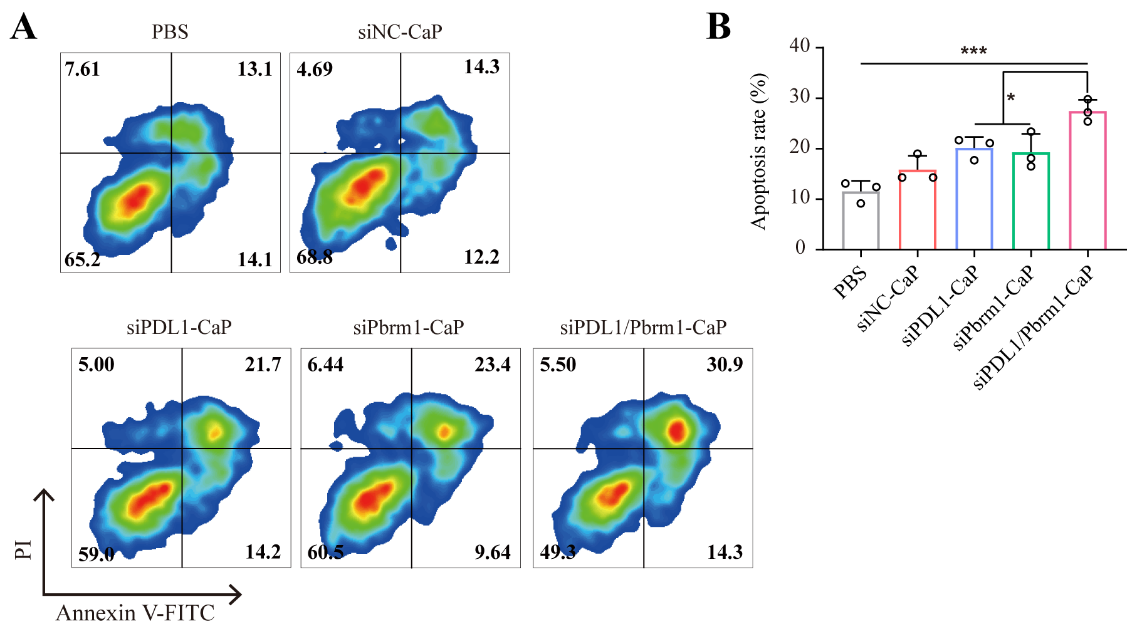


**Figure S24**. (A) Flow cytometry profiles of Hepa 1-6 cells with indicated treatments after co-incubation with CD8+T cells for 24 h using Annexin V-FITC/PI staining and (B) the statistical analysis of cell apoptotic rate. (*p < 0.05, ***p < 0.001, n=3).


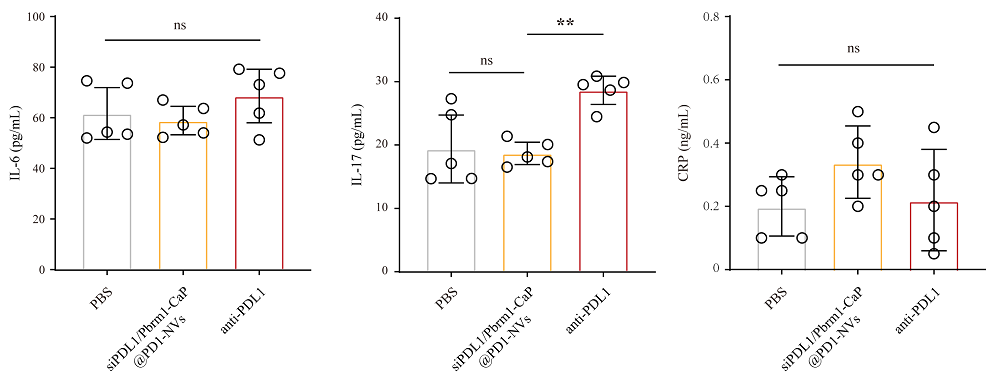


**Figure S25.** The cytokines (IL-6, IL-17) and C-reactive protein (CRP) levels in serum isolated from mice after receiving different treatment as indicated. (**p < 0.01, ***p < 0.001, ****p < 0.0001, n=5).


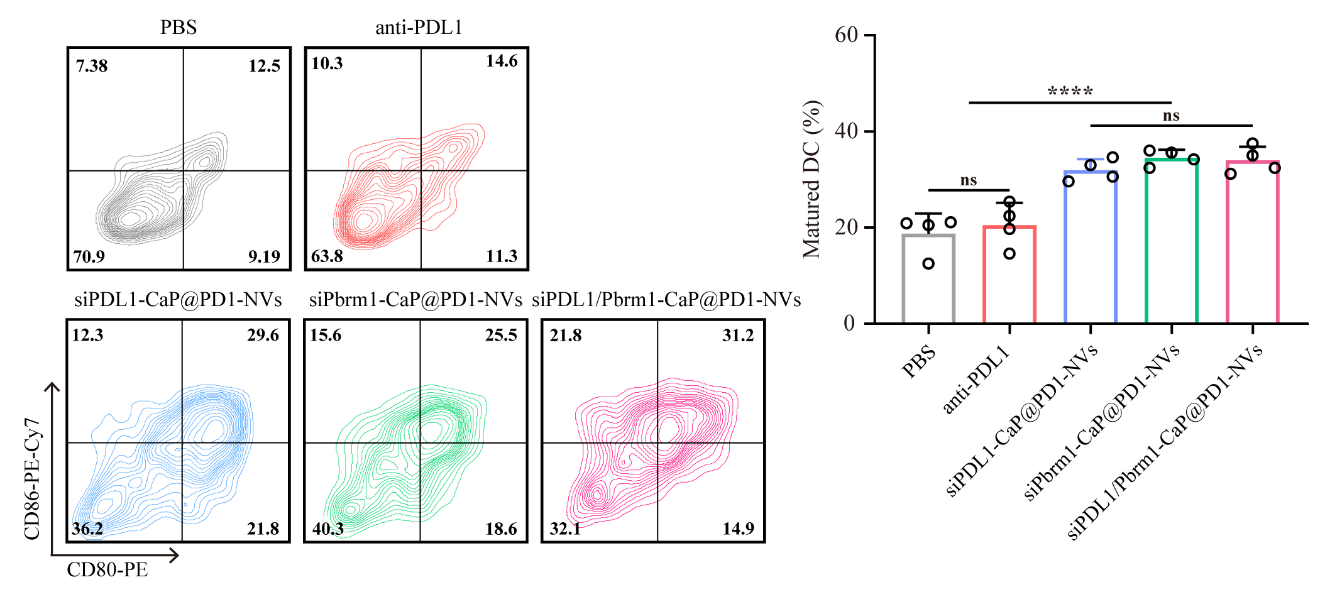


**Figure S26**. Flow cytometry analysis of the matured DC cells ratio in the spleen of mice in different treatment groups at the day 80. (ns represents not significant, ****p < 0.0001, n=4).


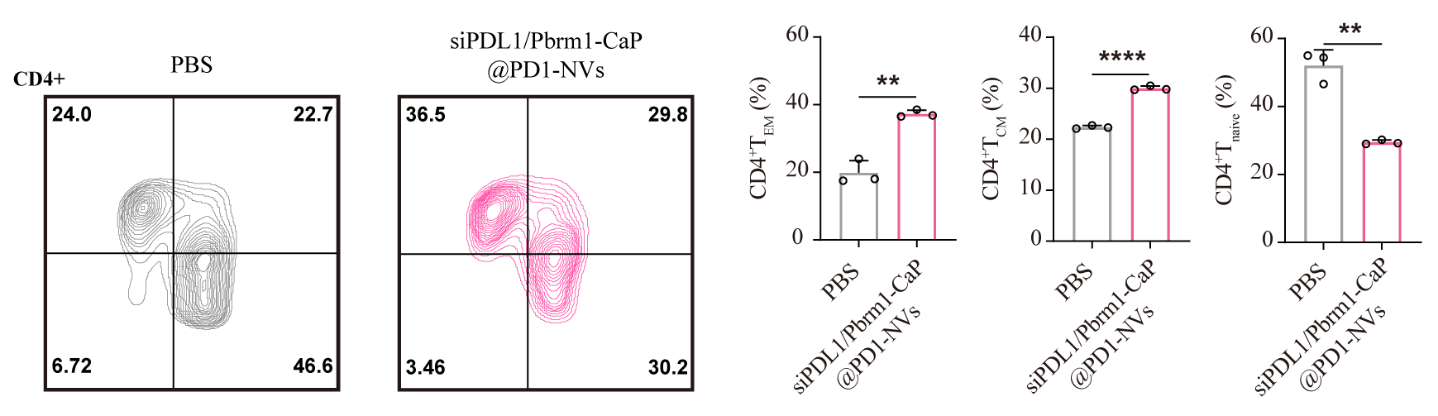


**Figure S27**. Flow cytometry profiles of T_EM_, T_CM_, and T_Naive_ in CD4+ T cells in the spleen of orthotopic Hepa1-6-luc bearing mice in different treatment groups at the day 80. (*p < 0.05, **p < 0.01, ****p < 0.0001, n=3)


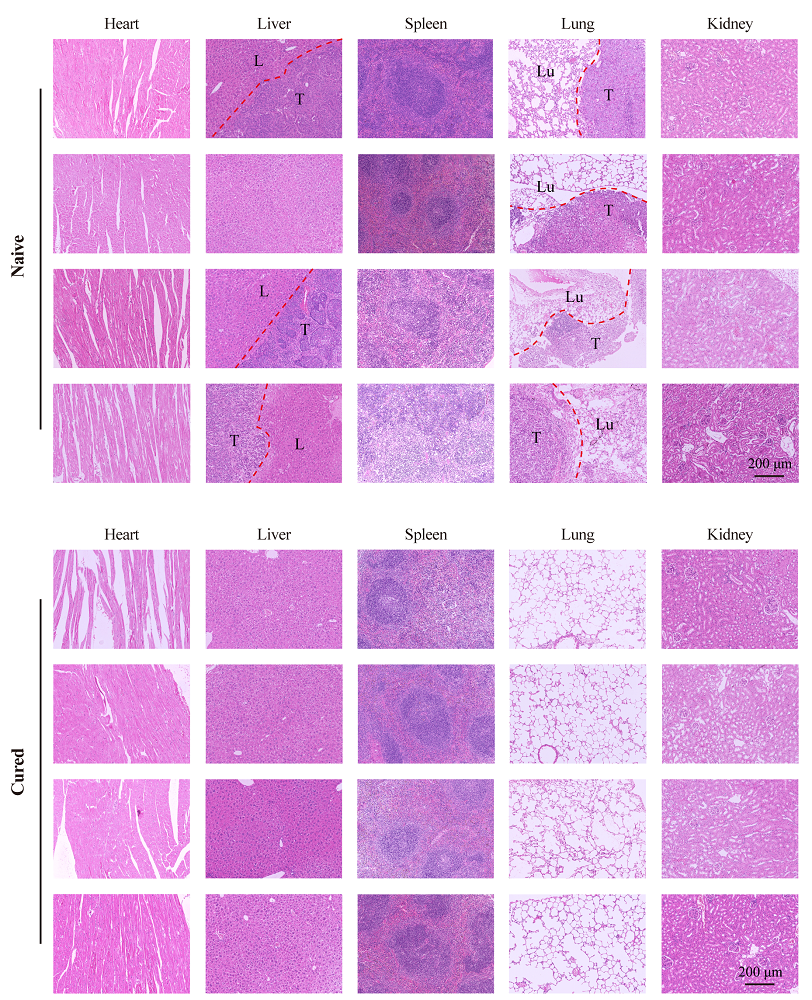


**Figure S28**. H&E staining of major organs isolated from the naive and cured mice after re-challenge as indicated. T indicated tumor tissues, L indicated liver tissues, and Lu indicated lung tissues. Scale bar, 200 μm.


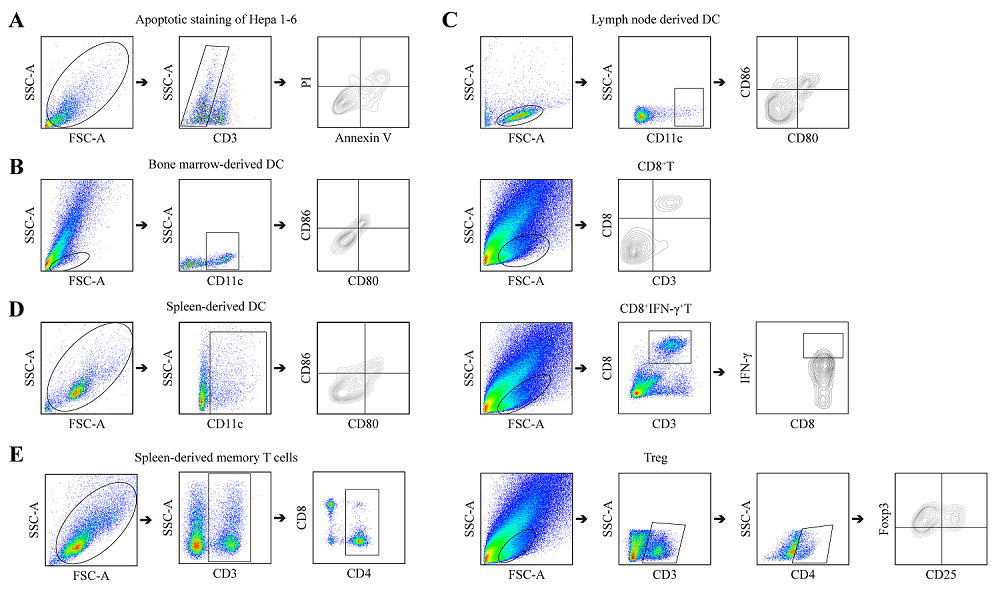


**Figure S29**. FACS gating strategy. (A) Apoptosis rate of Hepa 1-6 cells in Fig. 2D. (B) The ratio of CD80 and CD86 on bone marrow-derived DC cells in Fig. 2F. (C) The ratio of CD80 and CD86 on lymph nodes-derived DC cells and the ratio of CD8+ T cells, IFN-γ+ CD8+T cells and Treg cells in Fig. 4A-D. (D) The ratio of CD80 and CD86 on spleen-derived DC cells in Figure S26. (E) The spleen-derived memory T cells in Fig. 6D.

**Supplementary Tables**

**Table S1.** The sequences of siRNA

| **Name** | **Sequences (5`-3`)** |
| --- | --- |
| siPDL1 | Forward-GGAAAAGGAAGAUGAGCAATT  Reverse-UUGCUCAUCUUCCUUUUCCTT |
| siPbrm1 | Forward-GCUGAACCUUUCUUCCAUUTT  Reverse-AAUGGAAGAAAGGUUCAGCTT |
| siNC | Forward-UUCUCCGAACGUGUCACGUTT  Reverse-ACGUGACACGUUCGGAGAATT |

**Table S2.** The sequences of primers for RT-qPCR

| **Name** | **Sequences (5`-3`)** |
| --- | --- |
| β-actin | Forward-GTGACGTTGACATCCGTAAAGA  Reverse-GCCGGACTCATCGTACTCC |
| PDL1 | Forward-GCTCCAAAGGACTTGTACGTG  Reverse-TGATCTGAAGGGCAGCATTTC |
| Pbrm1 | Forward-TGACAGGTCCTTCGCACAATA  Reverse-TCTGATCCATACTGAAGTGCCA |
